# Supplementary material for: Proposals on Kaplan–Meier plots in medical research and a survey of stakeholder views: KMunicate
Source: BMJ Open. 2019 Sep 30;9(9):e030215. doi: 10.1136/bmjopen-2019-030215 (PMC6773317; doi:10.1136/bmjopen-2019-030215)

# 1 Supplementary information

## 1.1 Free text comments on survey (figure 19)

## 1.2 Email invitation text

Dear *name*

**Problem**
The standard way to present time-to-event data, such as survival, is with Kaplan–Meier plots. These are formatted by journals and reported in a number of ways, but we find they frequently lack some key information.
The key problems are:

1. Expressing how many people are contributing data at any point in the graph, including the pattern of censoring

2. Expressing that the uncertainty of the estimate increases over time

**Suggestion**
We have some initial suggestions on how to improve Kaplan–Meier plots, but we need your help to know which would be the most useful and most acceptable to a wide audience.

**Invitation to a short survey**
Could you take our short survey of nine meaningful multiple choice questions?

You will be asked to compare standard and alternative graphs, using data from one of three RCTS, chosen at random when you follow this link: bit.ly/KMunicate or http://www.ctu.mrc.ac.uk/resources/Kaplan-Meier/index.html.

Please complete the survey in one attempt as we cannot guarantee you will return to the same trial.

The survey will be open until **09-Jun-2017**.

**Survey results**
The findings will be written up for publication in a peer-reviewed journal and also introduced in an active poster session at the joint SCT & ICTMC 2017 conference in Liverpool.

We are interested to hear from anyone who looks at survival curves and are casting our net as wide as possible. Please forgive us if you have already received an invitation through another means.

If you have colleagues you think would be interested (including clinicians, journal editors, operations specialists, systematic reviewers, regulators, statisticians and trialists), please feel free to forward our invitation and link.

Thank you for your time.

Project team
Tim Morris, Chris Jarvis, Will Cragg, Babak Oskooei, Patrick Philips and Matt Sydes.



Morris TP, *et al. BMJ Open* 2019; 9:e030215. doi: 10.1136/bmjopen-2019-030215

## 1.3 Proposed alterations as presented to survey participants

The following supplementary figures are provided for readers to see the options we presented to survey participants and the descriptions from the survey. Figures 1, 2, 3, 4, 5 and 6 used data from the *RT01* trial; figures 7, 8, 9, 10, 11 and 12 used data from the *ICON7* trial; figures 13, 14, 15, 16, 17 and 18 used data from the *LY09* trial.

Figure 1: The extended at-risk table (*RT01* trial). The usual table beneath the plot contains the cumulative numbers censored by time $t$ and the cumulative number of events. Note that, at any time point, the three numbers sum to the number at risk at time 0.

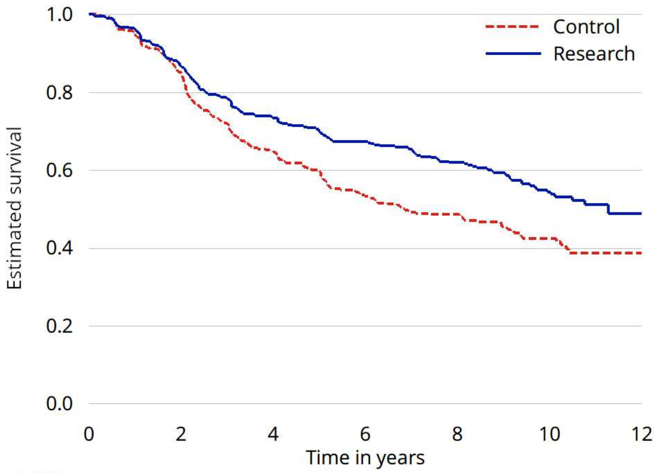


| | | | | | | | | | | | | | |
|---|---|---|---|---|---|---|---|---|---|---|---|---|---|
| **Control** | | | | | | | | | | | | | |
| At risk | 418 | 394 | 351 | 289 | 255 | 224 | 190 | 165 | 154 | 120 | 71 | 27 | 6 |
| Censored | 0 | 2 | 5 | 13 | 18 | 30 | 40 | 51 | 60 | 85 | 127 | 167 | 188 |
| Events | 0 | 22 | 62 | 116 | 145 | 164 | 188 | 202 | 204 | 213 | 220 | 224 | 224 |
| **Research** | | | | | | | | | | | | | |
| At risk | 418 | 397 | 351 | 313 | 283 | 263 | 239 | 218 | 198 | 165 | 99 | 39 | 8 |
| Censored | 0 | 5 | 12 | 17 | 27 | 35 | 48 | 62 | 71 | 96 | 151 | 208 | 237 |
| Events | 0 | 16 | 55 | 88 | 108 | 120 | 131 | 138 | 149 | 157 | 168 | 172 | 173 |



Morris TP, *et al. BMJ Open* 2019; 9:e030215. doi: 10.1136/bmjopen-2019-030215

Figure 2: At-risk lines (*RT01* trial). The usual table of numbers at risk is replaced by a line graph of the numbers at risk over time. It is effectively a less granular version but does not display the exact numbers at risk.

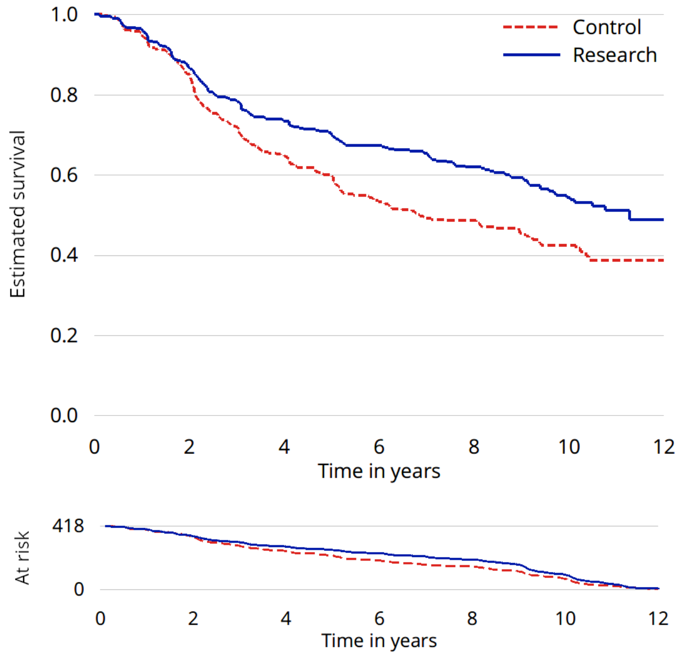




Morris TP, *et al. BMJ Open* 2019; 9:e030215. doi: 10.1136/bmjopen-2019-030215

Figure 3: At-risk areas beneath (*RT01* trial). This is a graphical form of the extended at-risk table. By arm, the cumulative number at risk, censored, and experiencing an event are given beneath the Kaplan–Meier plot.

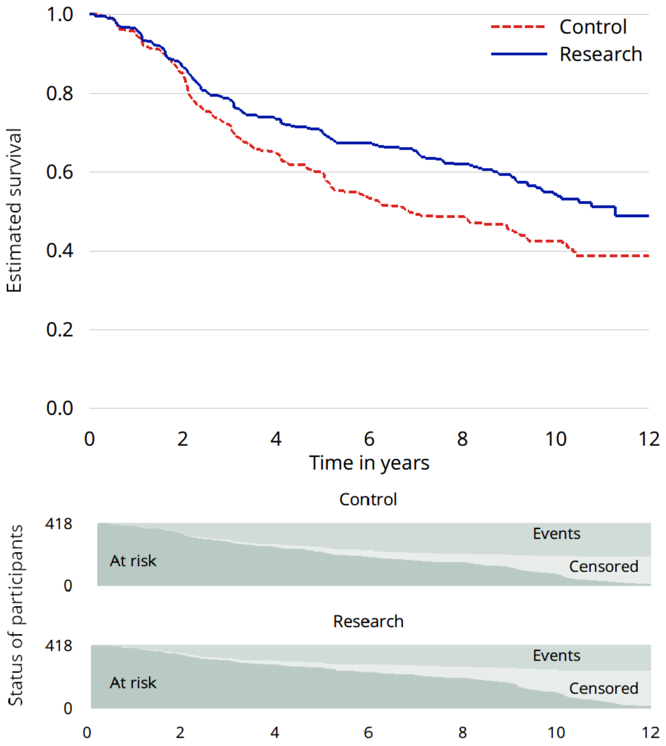




Morris TP, *et al. BMJ Open* 2019; 9:e030215. doi: 10.1136/bmjopen-2019-030215

Figure 4: At-risk areas behind the Kaplan–Meier plot (*RT01* trial). The graphical at-risk graphs are now drawn behind the Kaplan–Meier plot. Because there is one area graph for each arm, this necessitates repeating the Kaplan–Meier curves as many times as there are randomised arms.

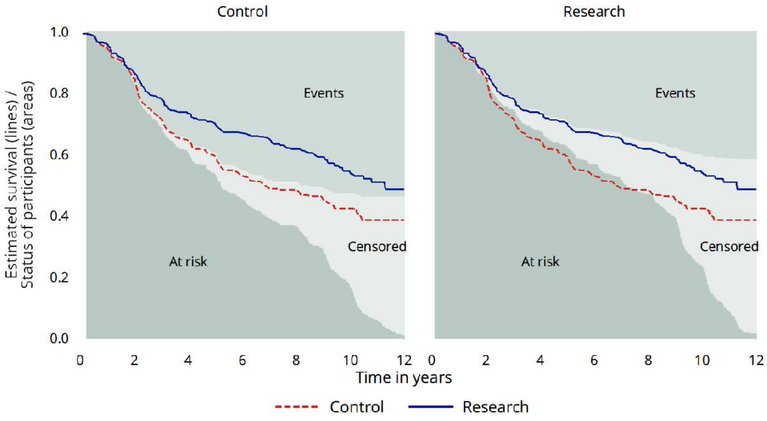




Morris TP, *et al. BMJ Open* 2019; 9:e030215. doi: 10.1136/bmjopen-2019-030215

Figure 5: Confidence intervals to depict uncertainty (*RT01* trial). Here, point-wise confidence intervals are plotted around the Kaplan–Meier estimate. We chose to plot these by shading of the area within the interval using the same colour as the line translucent, thus areas of overlap can be clearly seen.

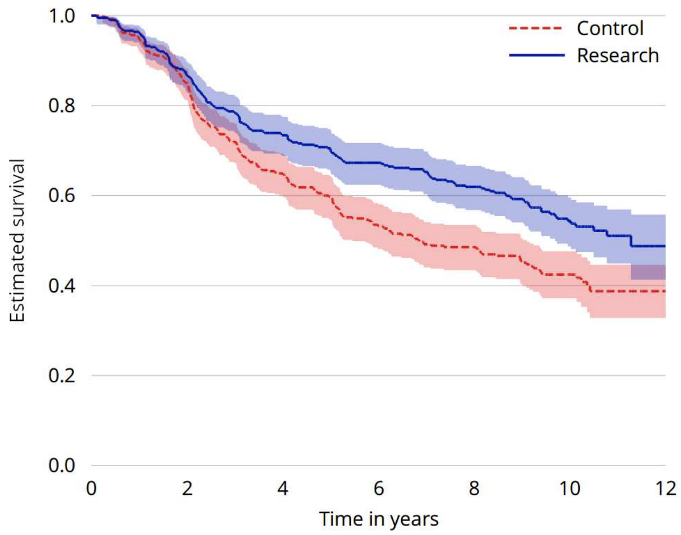




Morris TP, *et al. BMJ Open* 2019; 9:e030215. doi: 10.1136/bmjopen-2019-030215

Figure 6: Fading of the Kaplan–Meier estimates to depict uncertainty (*RT01* trial). Here, the curves fade in proportion to the cumulative number pf censored individuals (since it is censoring, not events, which means the estimate becomes more uncertain as time passes). The aim is to explicitly give the reader a visual deterrent when the eye is drawn to the far right.

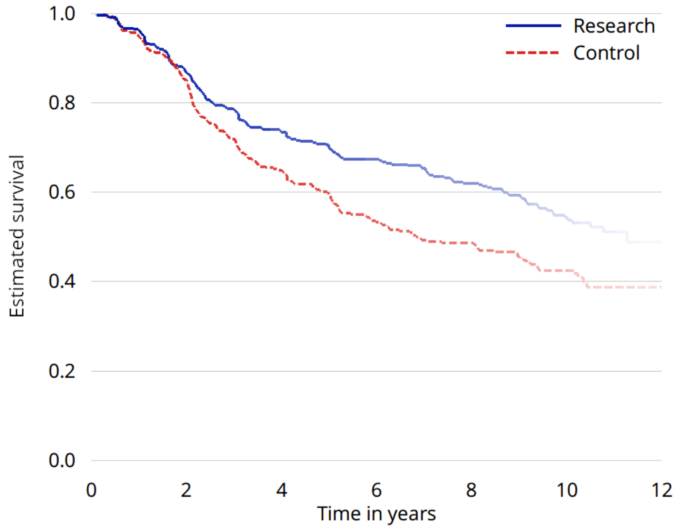




Morris TP, *et al. BMJ Open* 2019; 9:e030215. doi: 10.1136/bmjopen-2019-030215

Figure 7: The extended at-risk table (*ICON7* trial). The usual table beneath the plot contains the cumulative numbers censored by time $t$ and the cumulative number of events. Note that, at any time point, the three numbers sum to the number at risk at time 0.

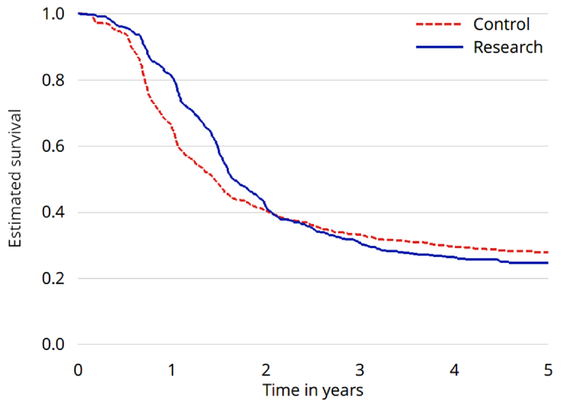


| | | | | | | | |
|---|---|---|---|---|---|---|---|
| **Control Arm** | At risk | 764 | 477 | 293 | 236 | 194 | 61 |
| | Events | 0 | 256 | 438 | 490 | 515 | 523 |
| | Censored | 0 | 31 | 33 | 38 | 55 | 180 |
| **Research Arm** | At risk | 764 | 600 | 306 | 224 | 177 | 49 |
| | Events | 0 | 143 | 434 | 514 | 545 | 553 |
| | Censored | 0 | 21 | 24 | 26 | 42 | 162 |

Figure 8: At-risk lines (*ICON7* trial). The usual table of numbers at risk is replaced by a line graph of the numbers at risk over time. It is effectively a less granular version but does not display the exact numbers at risk.

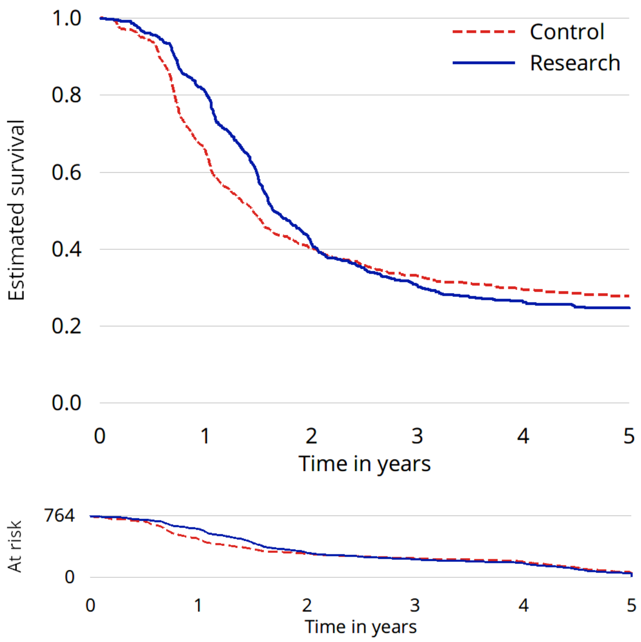




Morris TP, *et al*. *BMJ Open* 2019; 9:e030215. doi: 10.1136/bmjopen-2019-030215

Figure 9: At-risk areas beneath (*ICON7* trial). This is a graphical form of the extended at-risk table. By arm, the cumulative number at risk, censored, and experiencing an event are given beneath the Kaplan–Meier plot.

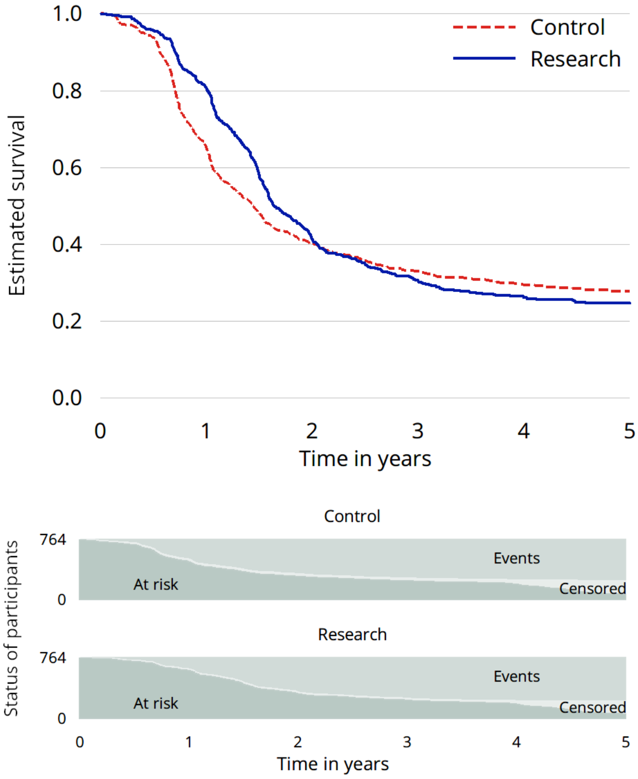




Morris TP, *et al*. *BMJ Open* 2019; 9:e030215. doi: 10.1136/bmjopen-2019-030215

Figure 10: At-risk areas behind the Kaplan–Meier plot (*ICON7* trial). The graphical at-risk graphs are now drawn behind the Kaplan–Meier plot. Because there is one area graph for each arm, this necessitates repeating the Kaplan–Meier curves as many times as there are randomised arms.

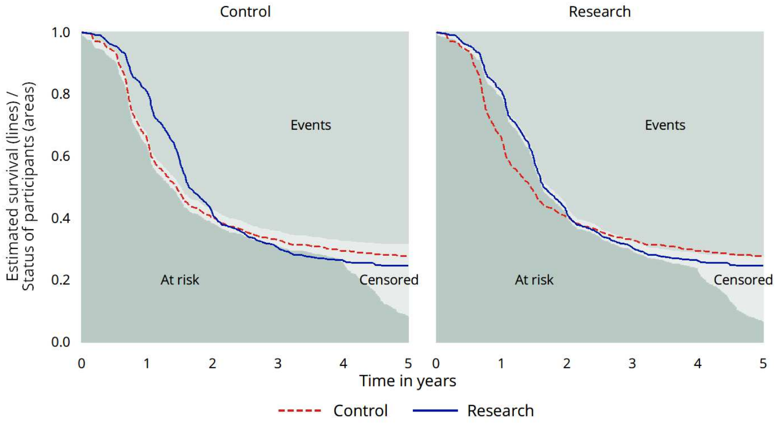




Morris TP, *et al. BMJ Open* 2019; 9:e030215. doi: 10.1136/bmjopen-2019-030215

Figure 11: Confidence intervals to depict uncertainty (*ICON7* trial). Here, point-wise confidence intervals are plotted around the Kaplan–Meier estimate. We chose to plot these by shading of the area within the interval using the same colour as the line translucent, thus areas of overlap can be clearly seen.

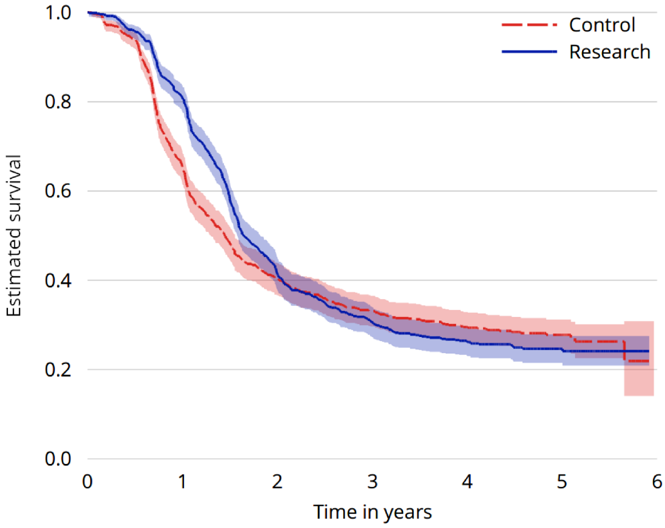




Morris TP, *et al*. *BMJ Open* 2019; 9:e030215. doi: 10.1136/bmjopen-2019-030215

Figure 12: Fading of the Kaplan–Meier estimates to depict uncertainty (*ICON7* trial). Here, the curves fade in proportion to the cumulative number of censored individuals (since it is censoring, not events, which means the estimate becomes more uncertain as time passes). The aim is to explicitly give the reader a visual deterrent when the eye is drawn to the far right.

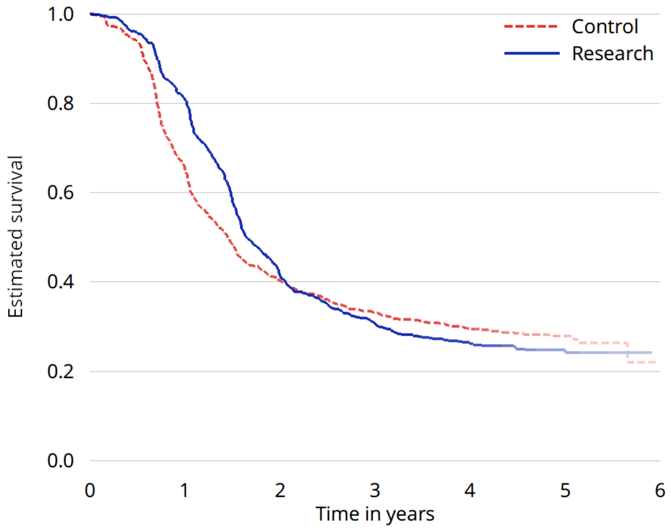




Morris TP, *et al*. *BMJ Open* 2019; 9:e030215. doi: 10.1136/bmjopen-2019-030215

Figure 13: The extended at-risk table (*LY09* trial). The usual table beneath the plot contains the cumulative numbers censored by time $t$ and the cumulative number of events. Note that, at any time point, the three numbers sum to the number at risk at time 0.

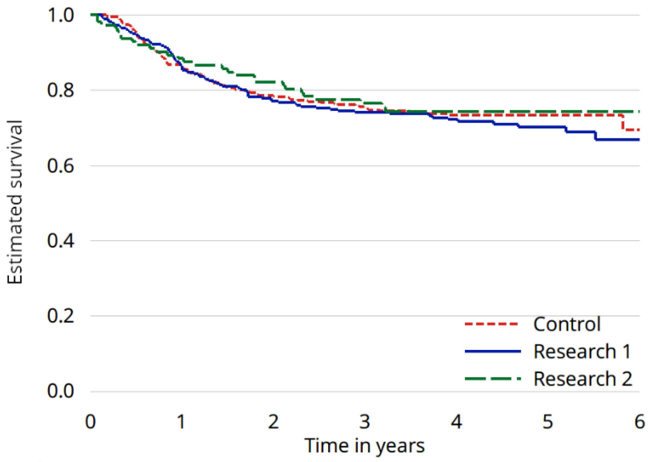


| | 0 | 1 | 2 | 3 | 4 | 5 | 6 |
|---|---|---|---|---|---|---|---|
| **Control** | | | | | | | |
| At risk | 394 | 338 | 302 | 265 | 178 | 79 | 10 |
| Censored | 0 | 3 | 7 | 33 | 114 | 213 | 281 |
| Events | 0 | 53 | 85 | 96 | 102 | 102 | 103 |
| **Research 1** | | | | | | | |
| At risk | 282 | 241 | 210 | 182 | 137 | 69 | 8 |
| Censored | 0 | 3 | 8 | 28 | 69 | 134 | 193 |
| Events | 0 | 38 | 64 | 72 | 76 | 79 | 81 |
| **Research 2** | | | | | | | |
| At risk | 112 | 99 | 90 | 75 | 52 | 25 | 3 |
| Censored | 0 | 0 | 2 | 11 | 32 | 59 | 81 |
| Events | 0 | 13 | 20 | 26 | 28 | 28 | 28 |



Morris TP, *et al. BMJ Open* 2019; 9:e030215. doi: 10.1136/bmjopen-2019-030215

Figure 14: At-risk lines (*LY09* trial). The usual table of numbers at risk is replaced by a line graph of the numbers at risk over time. It is effectively a less granular version but does not display the exact numbers at risk.

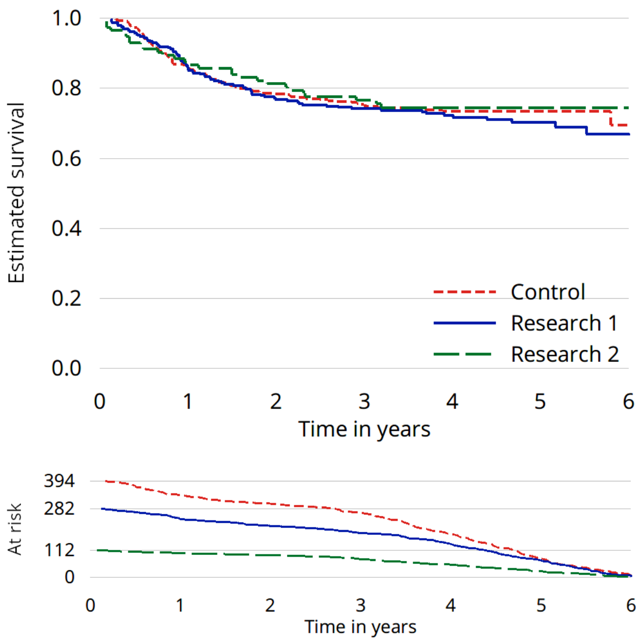




Morris TP, *et al. BMJ Open* 2019; 9:e030215. doi: 10.1136/bmjopen-2019-030215

Figure 15: At-risk areas beneath (*LY09* trial). This is a graphical form of the extended at-risk table. By arm, the cumulative number at risk, censored, and experiencing an event are given beneath the Kaplan–Meier plot.

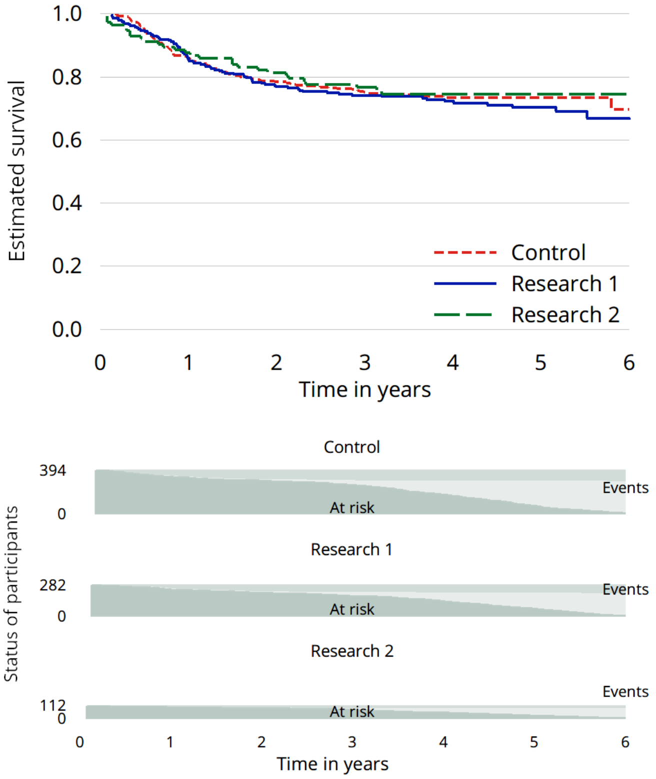




Morris TP, *et al. BMJ Open* 2019; 9:e030215. doi: 10.1136/bmjopen-2019-030215

Figure 16: At-risk areas behind the Kaplan–Meier plot (*LY09* trial). The graphical at-risk graphs are now drawn behind the Kaplan–Meier plot. Because there is one area graph for each arm, this necessitates repeating the Kaplan–Meier curves as many times as there are randomised arms.

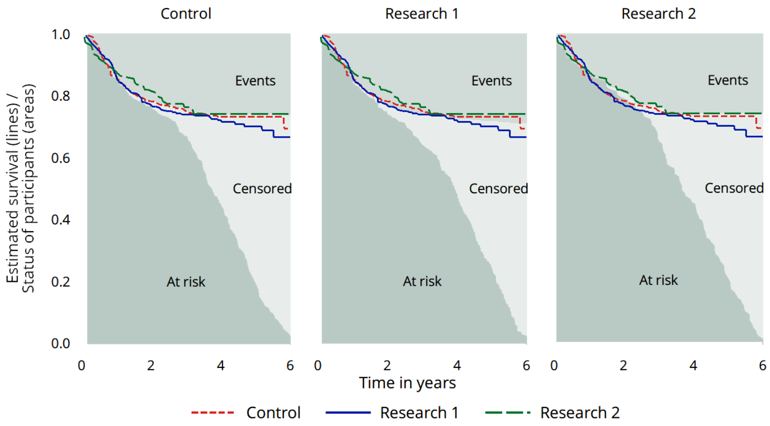




Morris TP, *et al. BMJ Open* 2019; 9:e030215. doi: 10.1136/bmjopen-2019-030215

Figure 17: Confidence intervals to depict uncertainty (*LY09* trial). Here, point-wise confidence intervals are plotted around the Kaplan–Meier estimate. We chose to plot these by shading of the area within the interval using the same colour as the line translucent, thus areas of overlap can be clearly seen.

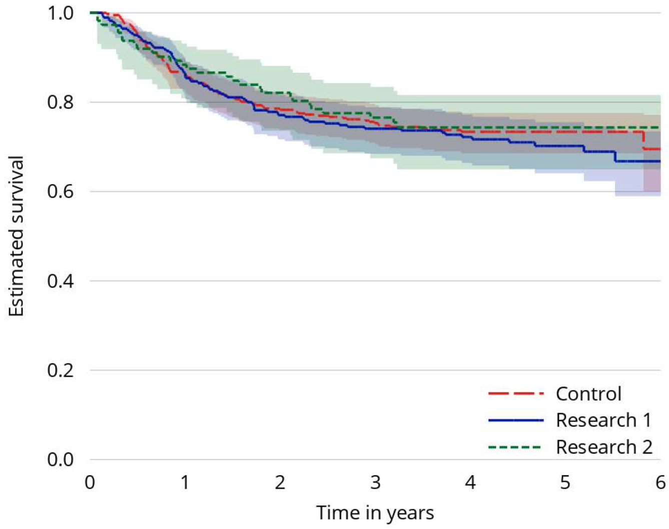




Morris TP, *et al. BMJ Open* 2019; 9:e030215. doi: 10.1136/bmjopen-2019-030215

Figure 18: Fading of the Kaplan–Meier estimates to depict uncertainty (*LY09* trial). Here, the curves fade in proportion to the cumulative number of censored individuals (since it is censoring, not events, which means the estimate becomes more uncertain as time passes). The aim is to explicitly give the reader a visual deterrent when the eye is drawn to the far right.

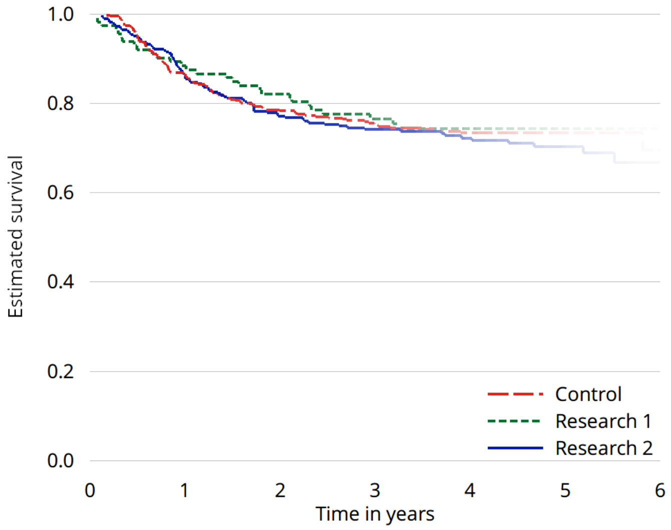




Morris TP, *et al. BMJ Open* 2019; 9:e030215. doi: 10.1136/bmjopen-2019-030215

### 1.4 Survey participants' experience with Kaplan–Meier

Figure 19: Years of experience 'reading and interpreting' *vs.* 'creating' Kaplan–Meier plots reported by participants. The margins give bar charts for 'reading and interpreting' (top) and 'creating' (right). For the bivariate plot, the top left indicates more time 'reading and interpreting' than 'creating' Kaplan–Meier plots.

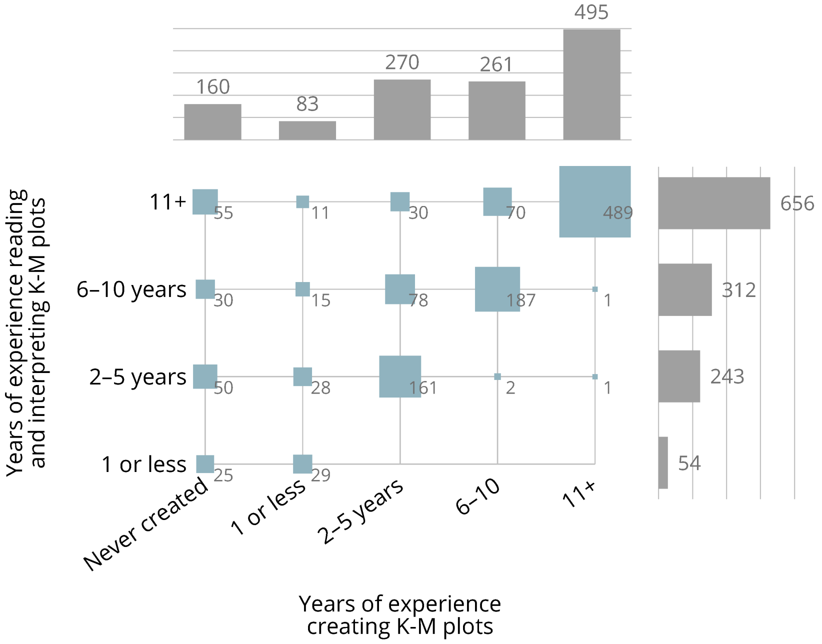




Morris TP, *et al. BMJ Open* 2019; 9:e030215. doi: 10.1136/bmjopen-2019-030215

Figure 20: Left: Summary of the nature of free-text comments (not mutually exclusive) on the specific candidate graphs; Right: Comments, suggestions and improvements, either specific to a graph or left as a general comment

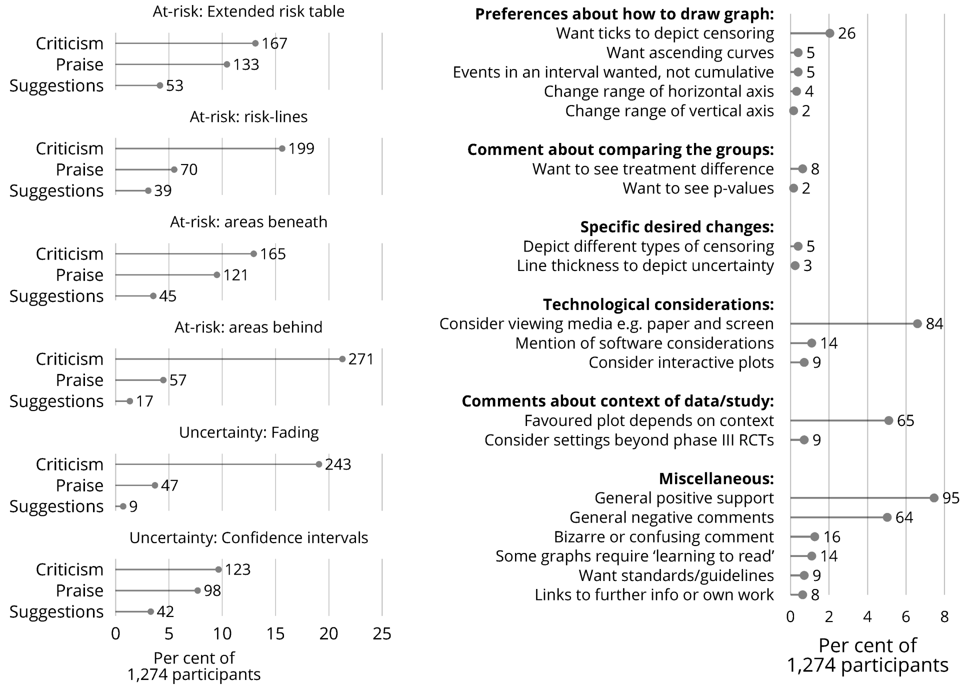

Supplement: Supplementary data [file bmjopen-2019-030215supp001.pdf]
