# Supplementary material for: Proposals on Kaplan–Meier plots in medical research and a survey of stakeholder views: KMunicate
Source: BMJ Open. 2019 Sep 30;9(9):e030215. doi: 10.1136/bmjopen-2019-030215 (PMC6773317; doi:10.1136/bmjopen-2019-030215)
Supplement: Author's manuscript [file bmjopen-2019-030215.draft_revisions.pdf]

BMJ Open is committed to open peer review. As part of this commitment we make the peer review history of every article we publish publicly available.

When an article is published we post the peer reviewers' comments and the authors' responses online. We also post the versions of the paper that were used during peer review. These are the versions that the peer review comments apply to.

The versions of the paper that follow are the versions that were submitted during the peer review process. They are not the versions of record or the final published versions. They should not be cited or distributed as the published version of this manuscript.

BMJ Open is an open access journal and the full, final, typeset and author-corrected version of record of the manuscript is available on our site with no access controls, subscription charges or pay-per-view fees (http://bmjopen.bmj.com).

If you have any questions on BMJ Open's open peer review process please email info.bmjopen@bmj.com

BMJ Open

Proposals on Kaplan–Meier plots in medical research and a survey of stakeholder views: KMunicate

Journal:	*BMJ Open*
Manuscript ID	bmjopen-2019-030215
Article Type:	Research
Date Submitted by the Author:	20-Mar-2019

Complete List of Authors:	Morris, Tim; MRC Clinical Trials Unit at UCL,<br>Jarvis, Christopher; London School of Hygiene and Tropical Medicine, Department of Infectious Disease Epidemiology<br>Cragg, William; University of Leeds, Clinical Trials Research Unit<br>Phillips, Patrick; University of California San Francisco, Division of Pulmonary and Critical Care Medicine<br>Choodari-Oskooei, Babak ; UCL , MRC Clinical Trials Unit at UCL<br>Sydes, Matthew; MRC Clinical Trials Unit at UCL,

Keywords:	Kaplan–Meier, Survival analysis, Data visualisation, Figures

SCHOLARONE™
Manuscripts

Proposals on Kaplan–Meier plots in medical research and a survey of stakeholder views: KMunicate

Tim P Morris*, https://orcid.org/0000-0001-5850-3610

Christopher I Jarvis, http://orcid.org/0000-0002-0812-2446

William Cragg, https://orcid.org/0000-0002-1274-8521

Patrick P J Phillips, https://orcid.org/0000-0002-6336-7024

Babak Choodari-Oskooei, https://orcid.org/0000-0001-7679-5899

Matthew R Sydes, https://orcid.org/0000-0002-9323-1371

MRC Clinical Trials Unit at UCL, Institute of Clinical Trials and Methodology, 90 High Holborn, London WC1V 6LJ. Tim Morris, Senior Research Associate in Medical Statistics; William Cragg, honorary research associate; Babak Choodari-Oskooei, Senior Research Associate in Medical Statistics; Matthew R Sydes, Reader in Clinical Trials.

Department of Infectious Disease Epidemiology, London School of Hygiene and Tropical Medicine, Keppel Street, London, WC1E 7HT. Christopher I Jarvis, Research Fellow.

Clinical Trials Research Unit, Leeds Institute of Clinical Trials Research, University of Leeds, Leeds, LS2 9JT. William Cragg, Regulatory and Governance Affairs Officer.

Division of Pulmonary and Critical Care Medicine, University of California San Francisco, 1001 Potrero Ave, San Francisco, CA 94110, USA. Patrick P J Phillips, Assistant Professor.

*Correspondence to Tim P Morris tim.morris@ucl.ac.uk

For peer review only

Contributor and guarantor information

The initial idea for this project came from Tim P Morris and Matthew R Sydes. All authors were involved in the initial planning, design and running of the study. Tim Morris and Chris Jarvis checked and cleaned the data and Tim Morris analysed the results. All authors commented critically on and approved the manuscript.

Tim Morris and Matthew Sydes are guarantors.

The corresponding author attests that all listed authors meet authorship criteria and that no others meeting the criteria have been omitted.

Copyright / license for publication

The Corresponding Author has the right to grant on behalf of all authors and does grant on behalf of all authors, a worldwide licence to the Publishers and its licensees in perpetuity, in all forms, formats and media (whether known now or created in the future), to i) publish, reproduce, distribute, display and store the Contribution, ii) translate the Contribution into other languages, create adaptations, reprints, include within collections and create summaries, extracts and/or, abstracts of the Contribution, iii) create any other derivative work(s) based on the Contribution, iv) to exploit all subsidiary rights in the Contribution, v) the inclusion of electronic links from the Contribution to third party material wherever it may be located; and, vi) licence any third party to do any or all of the above.

Competing interests declaration

All authors have completed the ICMJE uniform disclosure form at www.icmje.org/coi_disclosure.pdf and declare: no support from any organisation for the submitted work; no financial relationships with any organisations that might have an interest in the submitted work in the previous three years; no other relationships or activities that could appear to have in2uenced the submitted work.

Patient and public involvement

Patients were not involved with the development of the Kaplan–Meier proposals but were an important group of participants who were actively targeted by our survey. Alongside the researchers, 19 patients also participated.

Dissemination declaration

We will email results to all survey participants who stated that they wished to be updated. This will be done to coincide with publication of the manuscript.

For peer review only

Abstract

Objectives: To examine reactions to proposed improvements to standard Kaplan–Meier plots, the standard way to present time-to-event data, and to understand which (if any) facilitated better depiction of 1) the state of patients over time, 2) uncertainty over time in the estimates of survival.

Design: An opinion survey of stakeholders' opinions on the proposals.

Setting: A web-based survey, open to international participation, for those with an interest in visualisation of time-to-event data.

Participants: 1,174 people participated in the survey over a six-week period. Participation was global, (although primarily Europe and North America) and represented a wide range of researchers (primarily statisticians and clinicians).

Main outcome measures: Two outcome measures were of principal importance: 1) Participants' opinions of each proposal compared with a 'standard' Kaplan–Meier plot; 2) Participants overall ranking of the proposals (including the standard).

Results: Most proposals were more popular than the standard Kaplan–Meier plot. The most popular proposals in the two categories respectively were an extended table beneath the plot depicting the numbers at-risk, censored, and having experienced an event at periodic time points; and confidence intervals around each Kaplan–Meier curve.

Conclusions: This study produced a high response number, reflecting the importance of graphics for time-to-event data. Those producing and publishing Kaplan–Meier plots – both authors and journals – should, as a starting point, consider using the combination of the two favoured proposals. This should define a new standard against which future potential improvements can be tested for acceptability.

For peer review only

Article summary

Strengths and limitations of this study:

1. This study made several proposals to improve the information conveyed by Kaplan–Meier plots for survival data. Unlike many proposals for graphics, the study involved a survey of stakeholders' opinions.
2. A total of 1,174 people participated in the survey representing diverse professions, geographical locations and amounts of experience.
3. As a web-based survey for which participants selected themselves, it is not possible to know the number that might have participated and therefore the response proportion for this survey is unknown.

For peer review only

Introduction

Kaplan–Meier plots are ubiquitous in medical research, depicting the estimated cumulative proportion of people surviving over time. [1] This is sometimes presented overall, but frequently within groups, such as randomised arms of a clinical trial. For a clear and simple description of how the Kaplan–Meier estimate is calculated, see Bland and Altman. [2] In producing even a simple Kaplan–Meier plot, there are many choices to be made, leading to wide variation in presentation quality.

Figure 1 gives one example of a Kaplan–Meier plot (based on data from the RT01 trial) [3]. Box 1 outlines the basic anatomy of a Kaplan–Meier plot and highlights some of the choices to be made for readers who are unfamiliar.

Box 1: Anatomy of a Kaplan–Meier plot

In figure 1, the vertical axis runs from 0 to 1 and the horizontal from 0 to 12 years post-randomisation (though this was not the longest follow-up available). The Kaplan–Meier estimate for the control arm is depicted by a red dashed line and for the research arm by a solid blue line. The 'curves' are stepped over time because the estimate changes only at times when an event has occurred. These steps become more pronounced over time as more participants are censored. Beneath the horizontal axis is a table that reports the number of participants still 'at-risk' at specific time points (here 0, 2, 4, 6, 8, 10 and 12 years) *i.e.* they are still in follow-up at this time point, not having had an event or been censored. In figure 1, after 10 years, there remain 71 and 99 participants at risk of an event in the control and research arms respectively.

The utility of a Kaplan–Meier plot depends on who is using it and their purpose. Potential users may be: members of data monitoring committees considering interim data; systematic reviewers extracting data for meta-analysis; trial designers looking for information from relevant patients for sample size calculations; and clinicians trying to understand and communicate survival to their patients. Even when produced with care, key information may still be lacking for certain readers. It may seem that Kaplan–Meier plots do not require much 'learning to read'. However, we have many times been asked by collaborators how to read them.

We can learn a lot from a Kaplan–Meier plot: the estimated survival fraction at various times; the difference in survival fractions between two groups; quantiles of survival time; suggestions that hazard functions may be non-proportional. [4] It is even possible to reconstitute (data similar to) the underlying survival data based on Kaplan–Meier plots, often very accurately. [5]

Despite the above strengths, there are many issues which may hinder interpretation of Kaplan–Meier plots. The two key factors, and the focus of this work, are to communicate clearly:

1. the number of participants at-risk, censored, and having experienced an event at specific times, over time;
2. the uncertainty of the Kaplan–Meier estimate over time.

These aspects could aid interpretation and increase the amount of information conveyed by Kaplan–Meier plots. We believe a central problem is that a standard Kaplan-Meier plot does not clearly show that the right-hand portion of the curve (at later time points when there are usually considerably fewer patients at risk) is estimated with much greater uncertainty than the left-hand portion of the curve. As

For peer review only - http://bmjopen.bmj.com/site/about/guidelines.xhtml

a consequence, we are concerned that many consumers of Kaplan-Meier curves place undue emphasis on differences between curves at these later time points when differences are much more likely due to chance. ‘.’

As a snapshot describing recent practice, we reviewed the Kaplan–Meier plots presented in articles published in the BMJ, JAMA, The Lancet and NEJM during 2013. In total, there were 50 randomised, superiority trials with a time-to-event primary outcome. The two dominant specialities were cardiovascular disease (22 trials, 44%) and cancer (11 trials, 22%). Forty seven plots (94%) included a table of the numbers at risk over time, 10 (20%) depicted censoring in some way, either within a table beneath the plot or as ticks on the lines, and five (10%) depicted uncertainty using some form of confidence interval.

The objectives of this work are first, to identify alternatives in relation to the above issues, and second, to understand which (if any) alternatives offer improvements to standard practice.

Methods

Resulting directly from the objectives, the two activities undertaken were:

1. To propose some improvements in Kaplan–Meier plots; and
2. To survey stakeholders in order to understand which are preferred.

Graph development

The constraint on the first activity was that any proposals should still principally contain a figure showing the Kaplan–Meier estimate over time and should not be based on a different visual description of survival data (such as those in [6] and [7]).

A number of proposals were conceptualised, created and triaged. These were taken forward on the basis of being reasonably different to one another and favoured by at least one of the authors. This resulted in six proposals to take to survey, including four alternative means of representing the numbers at risk and two means of representing uncertainty.

Sources of data and randomisation

With the aim of covering a range of scenarios, we created the proposals for three phase III randomised trials:

1. RT01: a two-arm trial in prostate cancer which showed a clear difference in biochemical progression-free survival; [3]
2. Icon7: a two-arm trial in ovarian cancer with crossing survival curves; [8]
3. *LY09* [9]: a three-arm trial in Hodgkin’s lymphoma with limited differences between the arms. [9]

Participants were invited to take a short survey of 13 questions relating to these proposals.

To avoid the repetition and burden of answering all questions for each of the three trials, participants were randomly assigned to see graphs for just one of the trials, using simple randomisation in a 1:1:1 ratio (via a JavaScript tool invoked when a participant clicked the link to take the survey). The purpose

For peer review only

of this randomisation was not to compare the randomised groups (as in a randomised trial) but to elicit opinions averaged over these three scenarios.

Survey overview

The survey asked for participants' opinions and preferences regarding the six alternatives as compared with a reference that we regard as a reasonably 'standard' Kaplan–Meier plot (similar to figure 1; note that opinions as to what constitutes 'standard' is subject to opinion). The options are shown in figure 2 (for the RT01 trial data, with 'standard' based on authors' consensus).

In order to understand which of these proposals were preferred by stakeholders, we conducted a survey using the Bristol Online Surveys system.

Taking the survey

Participants were shown each proposed graph and asked to score it on a five-point ordinal scale, against the reference graph (without the proposed alteration, similar to that in figure 1), with the reference and proposal options visible side-by-side. Participants were next asked to rank (in order) their top-three graphs. This was done separately for the proposals addressing the numbers-at-risk and those addressing uncertainty. Participants were next asked to rank (in order) up to three of their preferred graphs.

After each of the above questions, participants had an opportunity to provide free-text comments, and a further opportunity to provide general comments on the survey. This gave a chance to explain their ratings of graphs. All of the free-text comments were read and categorised by the authors, with participants' comments assigned completely at random to one of BCO, CIJ, MJS, TPM or WJC. These comments were categorised in two ways. First, many of the comments were categorised as being to criticise, praise or suggest improvements to one of the proposals (most proposal-specific comments fell into one of these categories). Secondly, we categorised further comments (not proposal-specific) according to the comments made.

Baseline information collected

As well as opinions, we collected some participant characteristics. For descriptive purposes, we collected the country in which participants are primarily based, the date on which the survey was taken, and the years of experience 1) 'reading and interpreting', and 2) 'creating' Kaplan–Meier graphs. To explore whether opinions varied according to two specific characteristics, we also asked what participants identified as their principal professional background and whether or not they currently act as a journal editor. We regard the latter as important because journals often specify styles for Kaplan–Meier plots (either in instructions to authors or during typesetting) and so editors may exert disproportionate influence over what appears in the literature.

Target sample size

Our target sample size was 500 participants. This was based mainly on pragmatism.

For peer review only

Recruiting participants

We recruited participants by publicising the survey through many channels: emails to colleagues and collaborators, Twitter, email lists such as AllStat and the ISCB list, clinical collaborators of the MRC Clinical Trials Unit at UCL, the UK Hubs for Trials Methodology Research (note that this list is non-exhaustive). As the survey ran, we noted the high proportion of participants whose primary role was statistician, and so targeted clinicians and systematic reviewers more purposefully.

Analysis of results

Analysis of the data is descriptive, generally depicting the frequency of specific responses in graphs. The data on which the analysis is based are provided in the supplementary file for readers to explore themselves, minus the date of survey, free-text comments and participant country.

Note on ‘sampling’

The survey did not have any formal sampling mechanism (or well-defined units of the population, or its size) and is a convenience sample. We targeted those that we view as users and/or creators of Kaplan–Meier plots and who we could reach, for example, registered clinical trials units in the UK, journal editors, and systematic reviewers.

Patient and public involvement

There was no formal patient and public involvement in this project. Our objective was to improve researchers’ understanding of Kaplan–Meier plots. The survey itself was an attempt to involve such researchers by asking for their views on our proposals.

Results

One thousand, two hundred and seventy four participants completed the survey between 26 Apr 2017 and 7 July 2017.

Figure 3 gives descriptive information about the participants: self-described primary role/training, country in which they primarily work, whether they act as a journal editor, and experience (i) reading/interpreting, (ii) producing Kaplan–Meier plots.

The most represented roles were statistician (727; 57%) and clinician (341; 27%). Several other groups were well represented (see figure 3), but the results will be dominated by the groups identifying themselves as statistician or clinician. One hundred and seventy (14%) respondents identified themselves as journal editors. Participants were based primarily in the UK and USA but 36% were based in other countries, representing all populated continents (see figure 3).

Participants’ opinions on the proposed alterations to Kaplan–Meier plots are given in figure 4. The upper panel, upper row contains the proposals for presenting how the number at risk changes over time; the upper panel, lower row for those depicting uncertainty. On the upper row, the extended risk-table garnered the most positive opinions, with 1,054 (83%) participants giving a positive response. Some (110 participants; 9%) found this extra information ‘less useful’. Using a line graph to depict the numbers at risk was not popular, with 574 (45%) 1nding this less useful than the usual table depicting

For peer review only

numbers at risk. The graph of areas to replace the extended risk-table divided opinion: while 347 (27%) found it less useful than a standard plot, 772 (61%) found it ‘a bit’, ‘somewhat’ or ‘much more’ useful. The same chart with the areas superimposed behind the Kaplan–Meier estimate was much less popular, with 720 (57%) finding it less useful than the usual plot (at this first exposure). The lower row shows ambivalence about the idea of faded lines: 545 (43%) found this less useful than a standard presentation, 195 (15%) had no preference and 534 (42%) found it more useful.

These results were broadly similar across the three trials, both for statisticians and clinicians, and for editors and non-editors of journals. Figures similar to the upper panel of figure 4, broken down by these groups, can be found in the supplementary file.

The lower panel of figure 4 gives participants’ overall rankings for alternative presentations of numbers at risk and depictions of uncertainty. Green bars depict the number of participants who ranked this graph as their first choice; orange as second, red as third, and grey not ranked (for proposals depicting uncertainty, although there were only three options, participants did not have to answer for all choices if, for example, they found only one option to be acceptable). These results agree well with those presented in the upper panel of figure 4. For presenting numbers at risk, an extended risk table is the clear favourite; for depicting uncertainty, confidence intervals was the first choice for over half the participants.

An idea of the nature of free text responses is provided in figure in the supplementary file, which summarises whether graph-specific comments were criticism, praise, or suggestions (left) and gives the broad types of comment (right).

Discussion

We have proposed several alterations to ‘standard’ Kaplan–Meier plots, specifically for the context of showing within-arm survival in randomised trials. The proposals were around two key aspects depicting: 1) the numbers at risk over time and 2) uncertainty.

We then surveyed users of Kaplan–Meier plots for their views on our proposals. Several garnered more positive opinions than the reference plot, and two came out as the overall favourites, although opinions were far from unanimous.

We do not make explicit recommendations here about which alterations should be used but encourage producers of Kaplan–Meier plots and those who in2uence them (journal editors and regulators) to consider their practice in light of these results. In particular, the plots including an extended table of numbers and confidence intervals seemed to be favoured by most participants. These can be used in combination without any clash, and we include an example with both aspects in figure 5, again using the RT01 data.

There is clear recognition that graphical representations of time-to-event data could be improved. Many free text responses noted context. Kaplan–Meier plots are used by: trial designers looking for previous information on a related group of patients; data monitoring committees viewing interim data; meta-analysts to extract data; and clinicians looking to understand and communicate risks to their patients. There is no one-size that fits all settings and producers of Kaplan–Meier plots need to make judicious choices according to their context.

For peer review only

The two proposals involving area graphs to depict the number at risk require some thought to understand and are not instantly readable; a graph which requires little ‘learning to read’ is perhaps desirable. These two proposals were broadly unpopular in the survey: Many commented that this depiction was confusing, but a minority who liked them said it took time to reach that conclusion. Prior to the survey, the authors had expected the KM curve superimposed on the area depicting numbers at risk to be more popular than they were. The results of the survey show the desirability of a graph that requires little ‘learning to read’ and also the importance of a large stakeholder survey to elicit representative preferences.

Depicting the numbers-at-risk using line charts below the Kaplan–Meier plot was also reasonably unpopular. Free text comments suggested three main reasons: 1) participants wanted specific numbers in preference to a general pattern; 2) the line looks similar to the line of the Kaplan–Meier estimate, leading to potential confusion; and 3) as we created and presented this option, the plot region for the numbers in follow-up used 1/3 the area of the plot region for the Kaplan–Meier estimate, which for some participants was inadequate – a poor choice on our part. This proportion would need to be reconsidered by anyone looking to use the approach.

For depicting uncertainty, fading the Kaplan–Meier estimates was unpopular. There were two principal reasons for this. Firstly, when printed, the fading could be confused for a printing error, rather than an intended effect. Secondly, it is not clear how to define the level of decreasing intensity that accurate reflects the readers’ perception of increasing uncertainty. A minor comment from some clinicians was the desire to be able to accurately read the estimate at a very late time point (note that the premise for use of fading was in part to prevent this where uncertainty is extremely high).

Further thought is required on visualising survival data, and new proposals would ideally be accompanied by studies on stakeholders’ opinions. We constrained this project to Kaplan–Meier plots with two or more groups. However, in a randomised trial we are interested in comparing arms and so want to visualise some estimate of the difference. Such visualisations may be a fruitful future direction.

As noted in the methods section, the trial datasets we used do not represent any true distribution of scenarios occurring in clinical trials. Rather, they represent a small variety of situations which can occur in randomised trials; for any change to Kaplan–Meier plots to be worthwhile, the impact of features such as non-inferiority, non-proportional hazards, more than two arms, and different allocation ratios should be assessed. Having said this, if a plot works well for two-arm trials but not three-arm trials, it may of course be used in that context.

We hope that this work will provoke those creating Kaplan–Meier plots to think carefully about how they can best convey the information, and that journal editors will consider their policies for rendering Kaplan–Meier plots. We will continue to consider alternatives and evaluate these in the future.

Acknowledgements

We are grateful to the 1,274 participants who provided their opinions, particularly those who pointed us to articles containing their own thoughts on Kaplan–Meier; to the ICON7, LY09 and RE01 trial teams and participants for the use of these data.

Funding

For peer review only

This work was supported by the Medical Research Council (grant numbers MC_UU_12023/21, MC_UU_12023/29 and MC_UU_12023/24) – TM, WC, PP, BCO, MS.

Conflicts of Interest

The authors declare that they have no conflicts of interest.

References

[1] S. J. Pocock, T. G. Travison and L. M. Wruck, "Figures in clinical trial reports: current practice & scope for improvement," *Trials,* vol. 8, p. 36+, 2007.

[2] J. M. Bland and D. G. Altman, "Survival probabilities (the Kaplan-Meier method)," *BMJ,* vol. 317, pp. 1572-1580, 1998.

[3] D. P. Dearnaley, G. Jovic, I. Syndikus, V. Khoo, R. A. Cowan, J. D. Graham, E. G. Aird, D. Bottomley, R. A. Huddart, C. C. Jose, J. H. L. Matthews, J. L. Millar, C. Murphy, J. M. Russell, C. D. Scrase, M. K. B. Parmar and M. R. Sydes, "Escalated-dose versus control-dose conformal radiotherapy for prostate cancer: long-term results from the MRC RT01 randomised controlled trial," *The Lancet Oncology,* vol. 15, pp. 464-473, 2014.

[4] I. Ford and J. Norrie, "The role of covariates in estimating treatment effects and risk in long-term clinical trials," *Statist. Med.,* vol. 21, pp. 2899-2908, 15 10 2002.

[5] P. Guyot, A. E. Ades, M. J. Ouwens and N. J. Welton, "Enhanced secondary analysis of survival data: reconstructing the data from published Kaplan-Meier survival curves," *BMC Medical Research Methodology,* vol. 12, p. 9+, 2012.

[6] J. Newell, J. W. Kay and T. C. Aitchison, "Survival ratio plots with permutation envelopes in survival data problems," *Computers in Biology and Medicine,* vol. 36, pp. 526-541, 2006.

[7] P. Royston, M. K. B. Parmar and D. G. Altman, "Visualizing Length of Survival in Time-to-Event Studies: A Complement to Kaplan Meier Plots," *JNCI Journal of the National Cancer Institute,* vol. 100, pp. 92-97, 2008.

[8] A. M. Oza, A. D. Cook, J. Pfisterer, A. Embleton, J. A. Ledermann, E. Pujade-Lauraine, G. Kristensen, M. S. Carey, P. Beale, A. Cervantes, T. W. Park-Simon, G. Rustin, F. Joly, M. R. Mirza, M. Plante, M. Quinn, A. Poveda, G. C. Jayson, D. Stark, A. M. Swart, L. Farrelly, R. Kaplan, M. K. B. Parmar and T. J. Perren, "Standard chemotherapy with or without bevacizumab for women with newly diagnosed ovarian cancer (ICON7): overall survival results of a phase 3 randomised trial," *The Lancet Oncology,* vol. 16, pp. 928-936, 2015.

[9] P. W. M. Johnson, M. R. Sydes, B. W. Hancock, M. Cullen, J. A. Radford and S. P. Stenning, "Consolidation Radiotherapy in Patients With Advanced Hodgkin's Lymphoma: Survival Data From the UKLG LY09 Randomized Controlled Trial (ISRCTN97144519)," *Journal of Clinical Oncology,* vol. 28, pp. 3352-3359, 2010.

For peer review only

Captions for figures

Figure 1. An example of a Kaplan–Meier plot from the RT01 trial

Figure 2. Proposed graphs using the RT01 data

Figure 3. Descriptive characteristics of participants. Dot chart showing roles, editorial responsibilities, and experience with Kaplan–Meier (% on horizontal axis; frequencies labelled directly)

Figure 4. Upper panel: Opinion of alteration vs. `standard' Kaplan–Meier plot. Upper row is for alterations in presenting numbers at risk; lower row is for alterations in depicting uncertainty. Lower panel: Participants' overall preferences for presenting numbers at risk (upper part) and depicting uncertainty (lower part)

Figure 5. The two most popular elements combined: confidence intervals and extended at-risk table

For peer review only

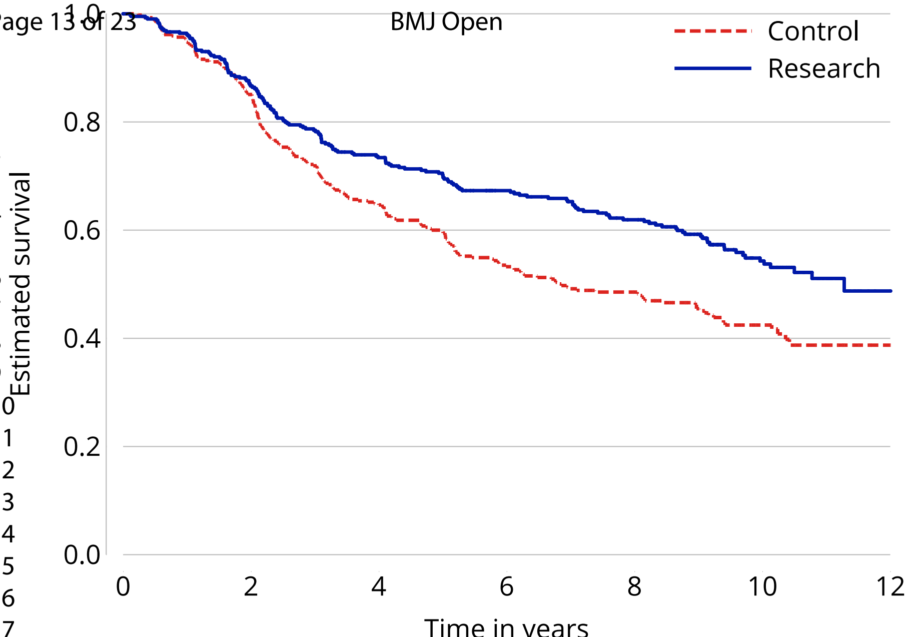

At risk

Control	418	351	255	190	154	71	6
Research	418	351	283	239	198	99	8

For peer review only - http://bmjopen.bmj.com/site/about/guidelines.xhtml

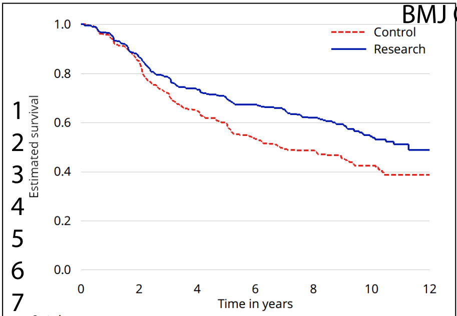

**Control**													
At risk	418	394	351	289	255	224	190	165	154	120	71	27	6
Censored	0	2	5	13	18	30	40	51	60	85	127	167	188
Events	0	22	62	116	145	164	188	202	204	213	220	224	224
**Research**													
At risk	418	397	351	313	283	263	239	218	198	165	99	39	8
Censored	0	5	12	17	27	35	48	62	71	96	151	208	237
Events	0	16	55	88	108	120	131	138	149	157	168	172	173

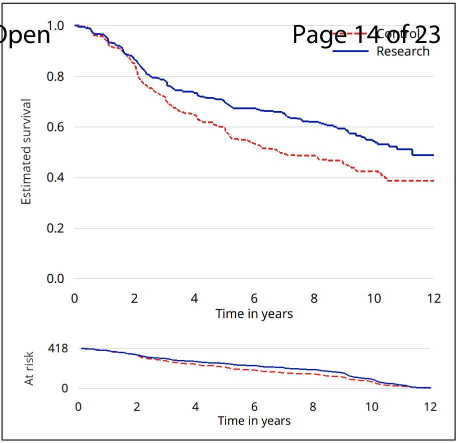

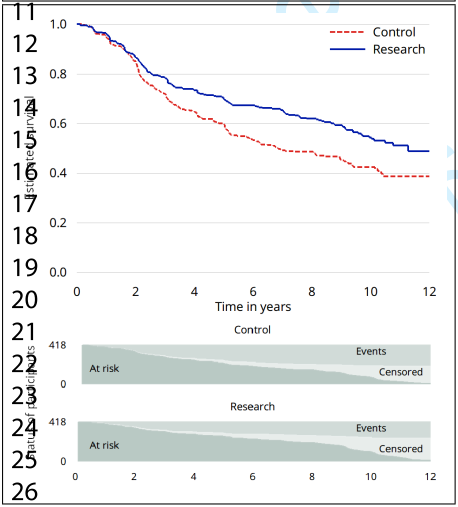

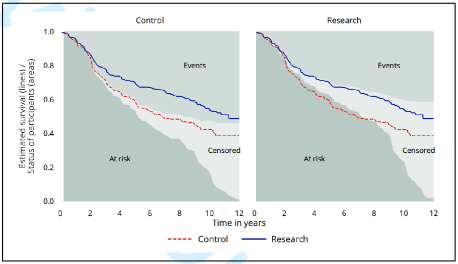

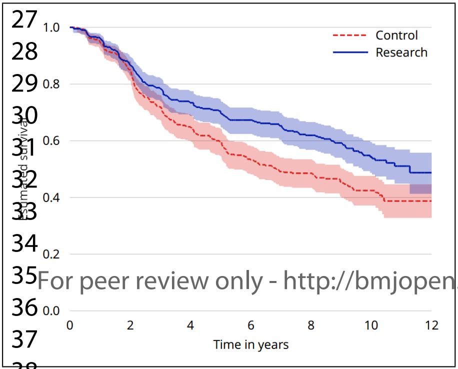

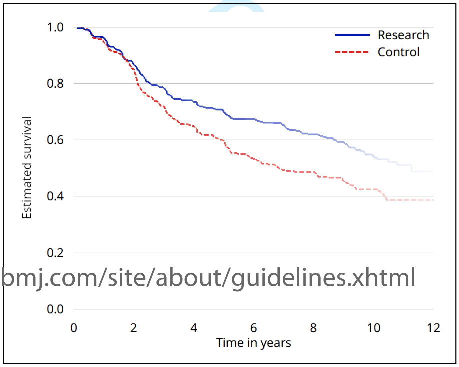

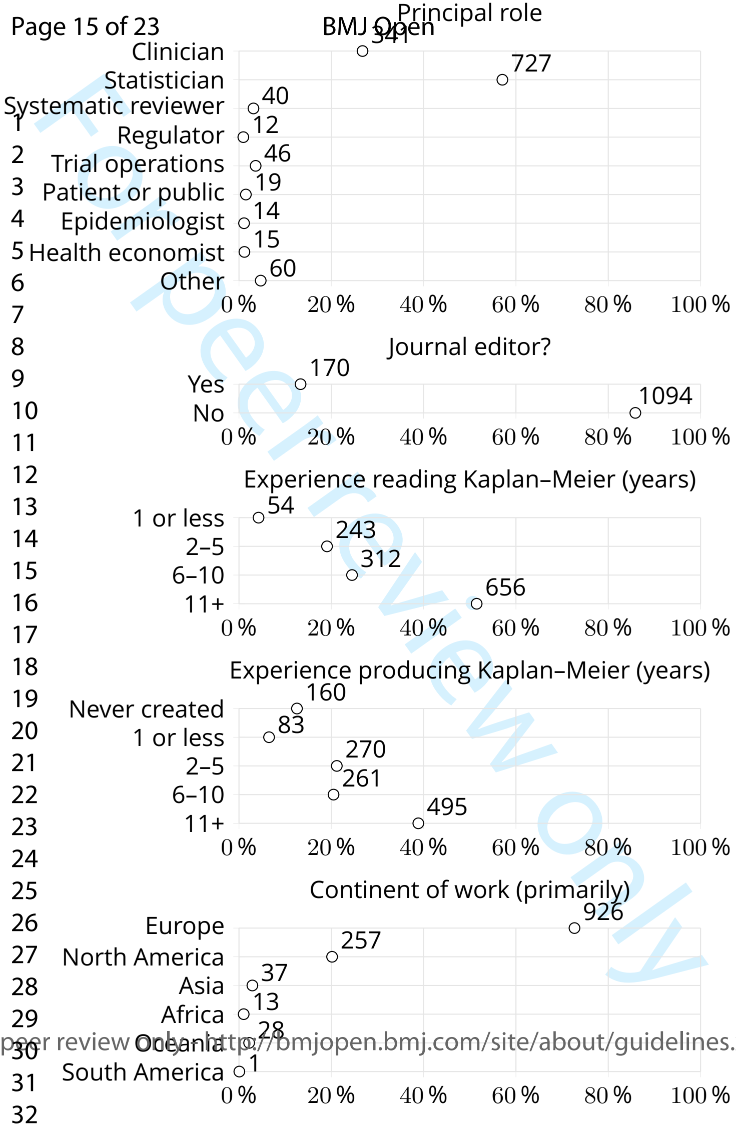

For peer review only - http://bmjopen.bmj.com/site/about/guidelines.xhtml

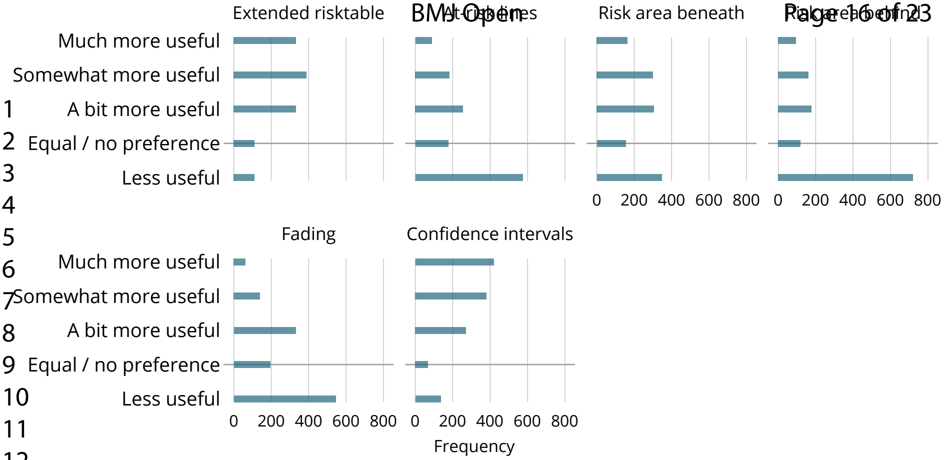

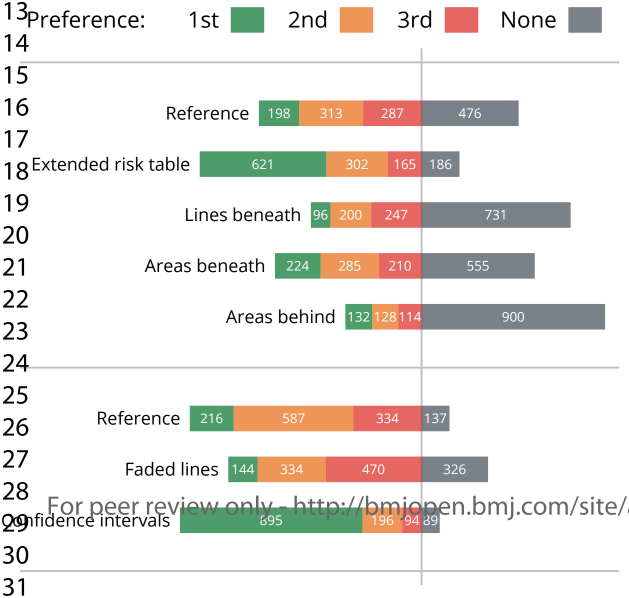

For peer review only - http://bmjopen.bmj.com/site/about/guidelines.xhtml

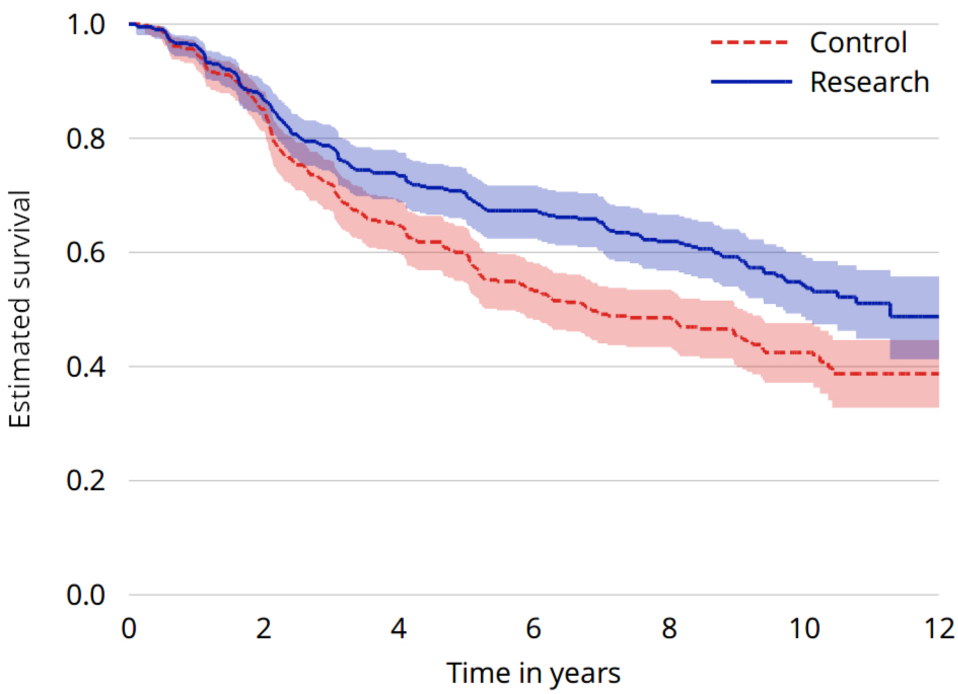

**Control**													
At risk	418	394	351	289	255	224	190	165	154	120	71	27	6
Censored	0	2	5	13	18	30	40	51	60	85	127	167	188
Events	0	22	62	116	145	164	188	202	204	213	220	224	224
**Research**													
At risk	418	397	351	313	283	263	239	218	198	165	99	39	8
Censored	0	5	12	17	27	35	48	62	71	96	151	208	237
Events	0	16	55	88	108	120	131	138	149	157	168	172	173

The two most popular elements combined: confidence intervals and extended at-risk table

1 Supplementary information

1.1 Free text comments on survey (figure 1)

Figure 1: Left: Summary of the nature of free-text comments (not mutually exclusive) on the specific candidate graphs; Right: Comments, suggestions and improvements, either specific to a graph or left as a general comment

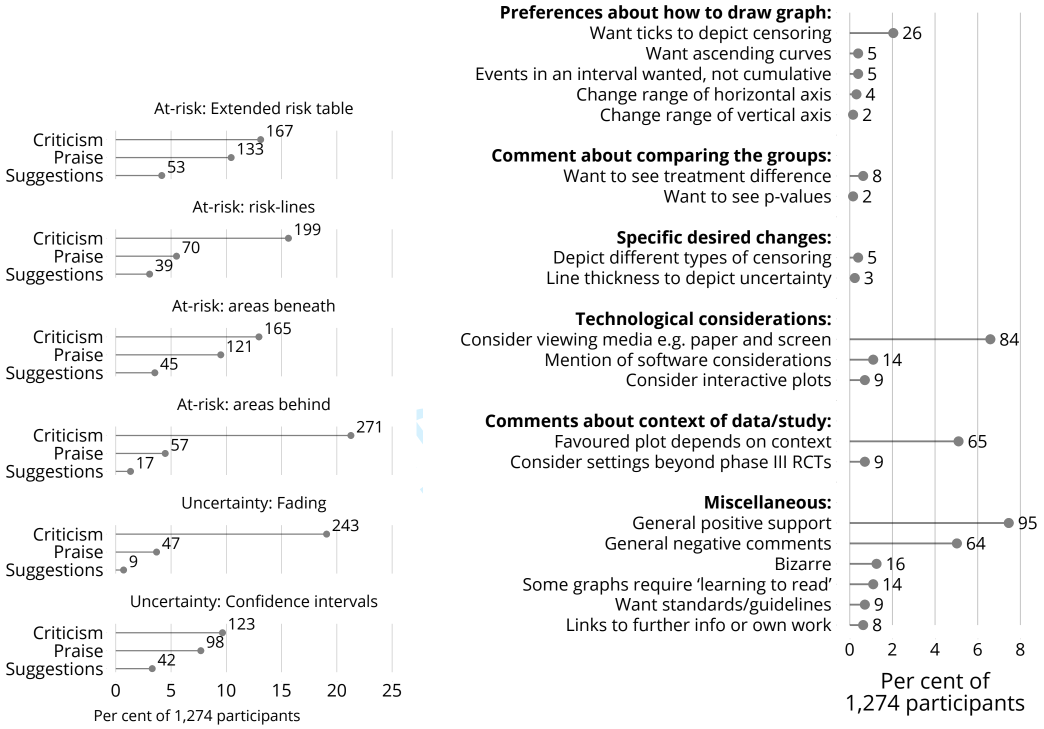

1.2 Proposed alterations for the *RT01* trial

The following supplementary figures (2, 3, 4, 5, 6, 7) are provided for readers to see the options we presented to survey participants and the descriptions from the survey.

1.3 Further results from survey

Figure 8 is included to describe the survey participants' experience with Kaplan–Meier plots.

1.4 Email invitation text

Dear *name*

Problem

For peer review only - http://bmjopen.bmj.com/site/about/guidelines.xhtml

The standard way to present time-to-event data, such as survival, is with Kaplan–Meier plots. These are formatted by journals and reported in a number of ways, but we find they commonly lack some key information.
The key problems are:

1. Expressing how many people are contributing data at any point in the graph, including the pattern of censoring

2. Expressing that the uncertainty of the estimate increases over time

Suggestion
We have some initial suggestions on how to improve Kaplan–Meier plots, but we need your help to know which would be the most useful and most acceptable to a wide audience.

Invitation to a short survey
Could you take our short survey of 9 meaningful multiple choice questions?

You'll be asked to compare standard and alternative graphs, using data from one of three RCTS, chosen at random when you follow this link: bit.ly/KMunicate or http://www.ctu.mrc.ac.uk/resources/Kaplan-Meier/index.html.

Please complete the survey in one attempt as we cannot guarantee you will return to the same trial.

The survey will be open until **09-Jun-2017**.

Survey results
The findings will be written up for publication in a peer-reviewed journal and also introduced in an active poster session at the joint SCT & ICTMC 2017 conference in Liverpool.

We are interested to hear from anyone who looks at survival curves and are casting our net as wide as possible. Please forgive us if you have already received an invitation through another means.

If you have colleagues you think would be interested (including clinicians, journal editors, operations specialists, systematic reviewers, regulators, statisticians and trialists), please feel free to forward our invitation and link.

Thank you for your time.

Project team

Tim Morris	MRC Clinical Trials Unit at UCL
Chris Jarvis	London School of Hygiene and Tropical Medicine
Will Cragg	MRC Clinical Trials Unit at UCL
Babak Oskooei	MRC Clinical Trials Unit at UCL
Patrick Philips	MRC Clinical Trials Unit at UCL
Matt Sydes	MRC Clinical Trials Unit at UCL

For peer review only - http://bmjopen.bmj.com/site/about/guidelines.xhtml

Figure 2: The extended at-risk table (*RT01* trial). The usual table beneath the plot contains the cumulative numbers censored by time t and the cumulative number of events. Note that, at any time point, the three numbers sum to the number at risk at time 0.

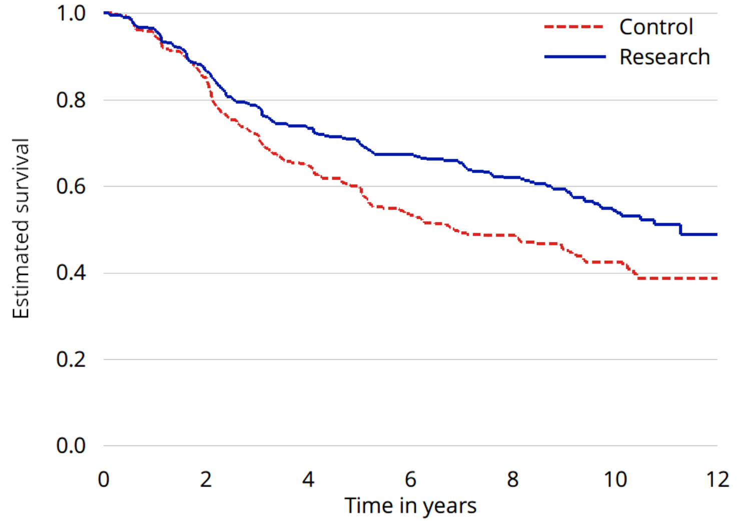

**Control**													
At risk	418	394	351	289	255	224	190	165	154	120	71	27	6
Censored	0	2	5	13	18	30	40	51	60	85	127	167	188
Events	0	22	62	116	145	164	188	202	204	213	220	224	224
**Research**													
At risk	418	397	351	313	283	263	239	218	198	165	99	39	8
Censored	0	5	12	17	27	35	48	62	71	96	151	208	237
Events	0	16	55	88	108	120	131	138	149	157	168	172	173

Figure 3: At-risk lines (*RT01* trial). The usual table of numbers at risk is replaced by a line graph of the numbers at risk over time. It is effectively a less granular version but does not display the exact numbers at risk.

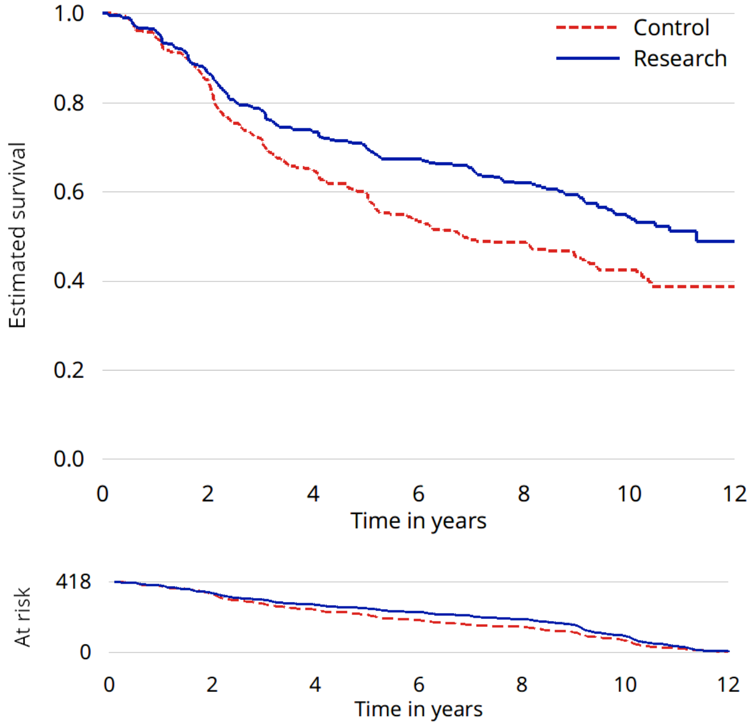

For peer review only - http://bmjopen.bmj.com/site/about/guidelines.xhtml

Figure 4: At-risk areas beneath (*RT01* trial). This is a graphical form of the extended at-risk table. By arm, the cumulative number at risk, censored, and experiencing an event are given beneath the Kaplan–Meier plot.

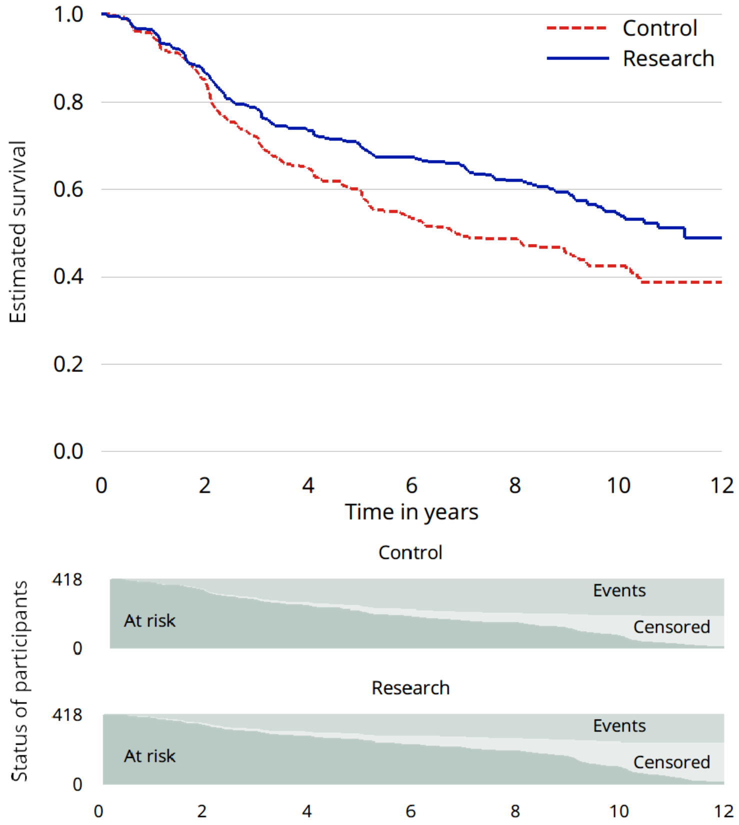

Figure 5: At-risk areas behind the Kaplan–Meier plot (*RT01* trial). The graphical at-risk graphs are now drawn behind the Kaplan–Meier plot. Because there is one area graph for each arm, this necessitates repeating the Kaplan–Meier curves as many times as there are randomised arms.

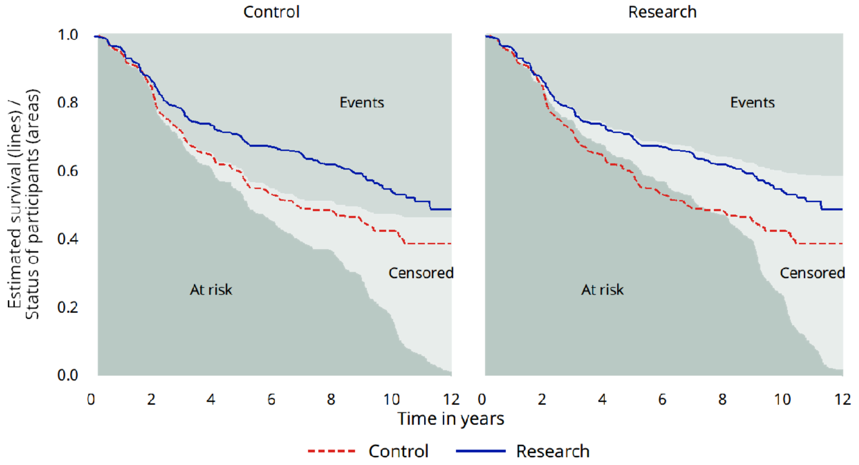

For peer review only - http://bmjopen.bmj.com/site/about/guidelines.xhtml

Figure 6: Confidence intervals to depict uncertainty (*RT01* trial). Here, point-wise confidence intervals are plotted around the Kaplan–Meier estimate. We chose to plot these by shading of the area within the interval using the same colour as the line translucent, thus areas of overlap can be clearly seen.

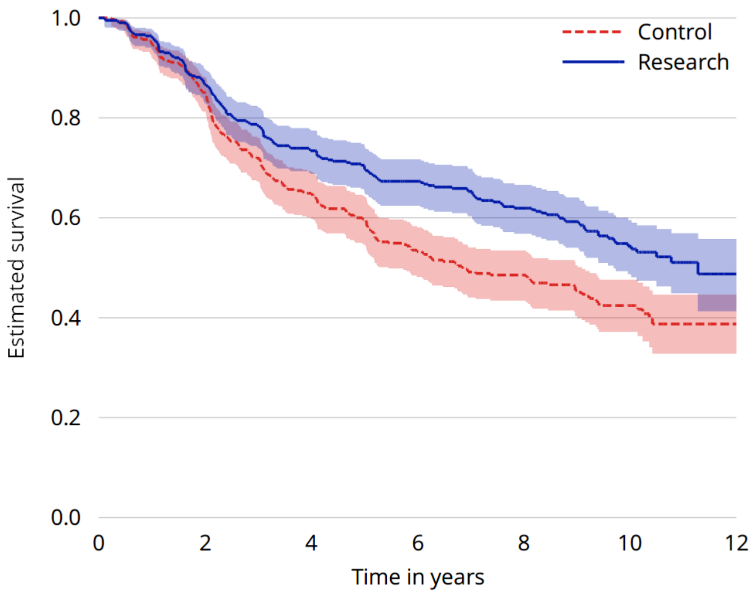

Figure 7: Fading of the Kaplan–Meier estimates to depict uncertainty (*RT01* trial). Here, the curves fade in proportion to the cumulative number f censored individuals (since it is censoring, not events, which means the estimate becomes more uncertain as time passes). The aim is to explicitly give the reader a visual deterrent when the eye is drawn to the far right.

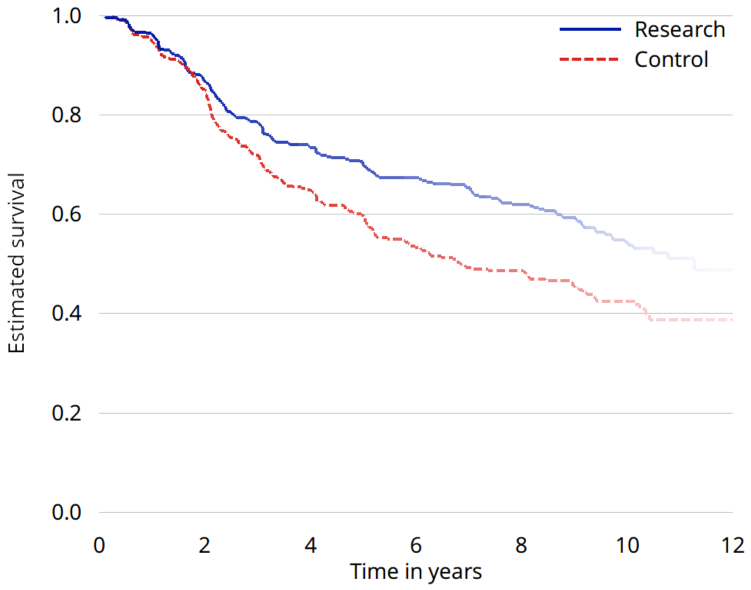

For peer review only - http://bmjopen.bmj.com/site/about/guidelines.xhtml

Figure 8: Years of experience 'reading and interpreting' *vs.* 'creating' Kaplan–Meier plots reported by participants. The margins give bar charts for 'reading and interpreting' (top) and 'creating' (right). For the bivariate plot, the top left indicates more time 'reading and interpreting' than 'creating' Kaplan–Meier plots.

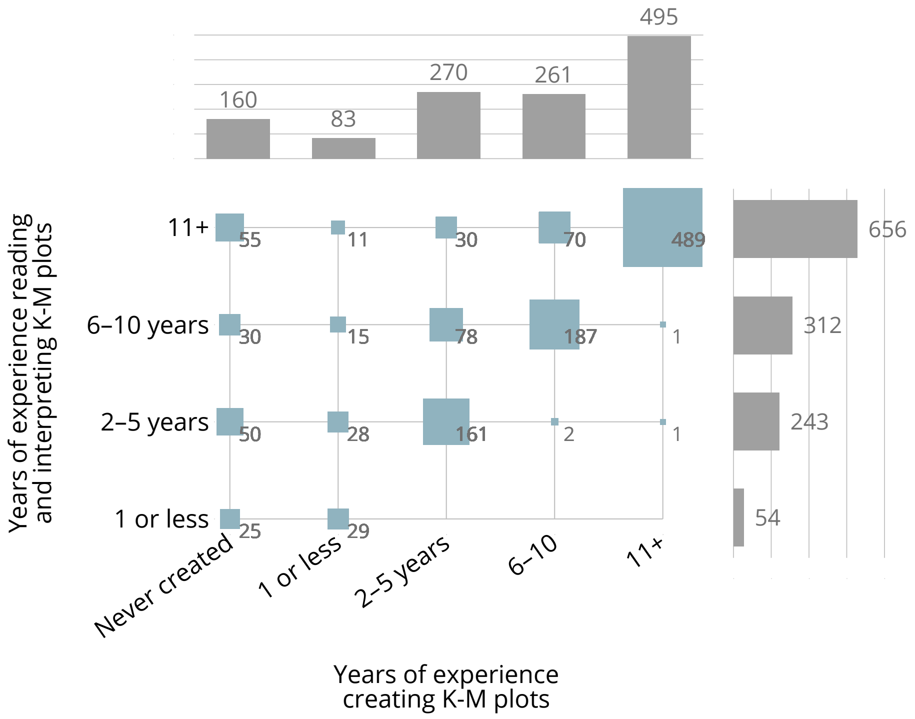

BMJ Open

Proposals on Kaplan–Meier plots in medical research and a survey of stakeholder views: KMunicate

Journal:	*BMJ Open*
Manuscript ID	bmjopen-2019-030215.R1
Article Type:	Research
Date Submitted by the Author:	30-Jul-2019
Complete List of Authors:	Morris, Tim; MRC Clinical Trials Unit at UCL,<br>Jarvis, Christopher; London School of Hygiene and Tropical Medicine, Department of Infectious Disease Epidemiology<br>Cragg, William; University of Leeds, Clinical Trials Research Unit<br>Phillips, Patrick; University of California San Francisco, Center for TB<br>Choodari-Oskooei, Babak ; UCL , MRC Clinical Trials Unit at UCL<br>Sydes, Matthew; MRC Clinical Trials Unit at UCL,
**Primary Subject Heading**:	Research methods
Secondary Subject Heading:	Oncology
Keywords:	Kaplan–Meier, Survival analysis, Data visualisation, Figures, Clinical trials < THERAPEUTICS, Graphs

SCHOLARONE™
Manuscripts

Proposals on Kaplan–Meier plots in medical research and a survey of stakeholder views: KMunicate

Tim P Morris*, https://orcid.org/0000-0001-5850-3610

Christopher I Jarvis, http://orcid.org/0000-0002-0812-2446

William Cragg, https://orcid.org/0000-0002-1274-8521

Patrick P J Phillips, https://orcid.org/0000-0002-6336-7024

Babak Choodari-Oskooei, https://orcid.org/0000-0001-7679-5899

Matthew R Sydes, https://orcid.org/0000-0002-9323-1371

MRC Clinical Trials Unit at UCL, Institute of Clinical Trials and Methodology, 90 High Holborn, London WC1V 6LJ. Tim Morris, Senior Research Associate in Medical Statistics; William Cragg, honorary research associate; Babak Choodari-Oskooei, Senior Research Associate in Medical Statistics; Matthew R Sydes, Reader in Clinical Trials.

Department of Infectious Disease Epidemiology, London School of Hygiene and Tropical Medicine, Keppel Street, London, WC1E 7HT. Christopher I Jarvis, Research Fellow.

Clinical Trials Research Unit, Leeds Institute of Clinical Trials Research, University of Leeds, Leeds, LS2 9JT. William Cragg, Regulatory and Governance Affairs Officer.

Center for TB, University of California San Francisco, 1001 Potrero Ave, San Francisco, CA 94110, USA. Patrick P J Phillips, Assistant Professor.

*Correspondence to Tim P Morris tim.morris@ucl.ac.uk

For peer review only

Contributor and guarantor information

The initial idea for this project came from Tim P Morris and Matthew R Sydes. All authors (Tim P Morris, Matther R Sydes, Christopher I Jarvis, Patrick P J Phillips, William Cragg and Babak Choodari-Oskooei) were involved in the initial planning, design and running of the study. Tim P Morris and Chris I Jarvis checked and cleaned the data and Tim P Morris analysed the results. All authors (Tim P Morris, Matther R Sydes, Christopher I Jarvis, Patrick P J Phillips, William Cragg and Babak Choodari-Oskooei) commented critically on and approved the manuscript.

Tim P Morris and Matthew R Sydes are guarantors.

The corresponding author attests that all listed authors meet authorship criteria and that no others meeting the criteria have been omitted.

Copyright / license for publication

The Corresponding Author has the right to grant on behalf of all authors and does grant on behalf of all authors, a worldwide licence to the Publishers and its licensees in perpetuity, in all forms, formats and media (whether known now or created in the future), to i) publish, reproduce, distribute, display and store the Contribution, ii) translate the Contribution into other languages, create adaptations, reprints, include within collections and create summaries, extracts and/or, abstracts of the Contribution, iii) create any other derivative work(s) based on the Contribution, iv) to exploit all subsidiary rights in the Contribution, v) the inclusion of electronic links from the Contribution to third party material wherever it may be located; and, vi) licence any third party to do any or all of the above.

Competing interests declaration

All authors have completed the ICMJE uniform disclosure form at www.icmje.org/coi_disclosure.pdf and declare: no support from any organisation for the submitted work; no financial relationships with any organisations that might have an interest in the submitted work in the previous three years; no other relationships or activities that could appear to have influenced the submitted work.

Patient and public involvement

There was no formal patient and public involvement in the development of the Kaplan–Meier proposals but patients were an important group of participants who were actively targeted by our survey. Alongside the researchers, 19 patients participated. Our objective was to improve researchers' understanding of Kaplan–Meier plots. The survey itself was an attempt to involve such researchers by asking for their views on our proposals.

Dissemination declaration

We will email results to all survey participants who stated that they wished to be updated. This will be done to coincide with publication of the manuscript.

For peer review only

Abstract

Objectives: To examine reactions to proposed improvements to standard Kaplan–Meier plots, the standard way to present time-to-event data, and to understand which (if any) facilitated better depiction of 1) the state of patients over time, 2) uncertainty over time in the estimates of survival.

Design: A survey of stakeholders' opinions on the proposals.

Setting: A web-based survey, open to international participation, for those with an interest in visualisation of time-to-event data.

Participants: 1,174 people participated in the survey over a six-week period. Participation was global, (although primarily Europe and North America) and represented a wide range of researchers (primarily statisticians and clinicians).

Main outcome measures: Two outcome measures were of principal importance: 1) Participants' opinions of each proposal compared with a 'standard' Kaplan–Meier plot; 2) Participants' overall ranking of the proposals (including the standard).

Results: Most proposals were more popular than the standard Kaplan–Meier plot. The most popular proposals in the two categories respectively were an extended table beneath the plot depicting the numbers at-risk, censored, and having experienced an event at periodic time points; and confidence intervals around each Kaplan–Meier curve.

Conclusions: This study produced a high response number, reflecting the importance of graphics for time-to-event data. Those producing and publishing Kaplan–Meier plots – both authors and journals – should, as a starting point, consider using the combination of the two favoured proposals.

For peer review only

Article summary

Strengths and limitations of this study:

1. This study made several proposals to improve the information conveyed by Kaplan–Meier plots for survival data. Unlike many proposals for graphics, the study involved a survey of stakeholders' opinions.
2. A total of 1,174 people participated in the survey representing diverse professions, geographical locations and amounts of experience.
3. As a web-based survey for which participants selected themselves, it is not possible to know the number that might have participated and therefore the response proportion for this survey is unknown.

For peer review only

Introduction

Kaplan–Meier plots are ubiquitous in medical research, depicting the estimated cumulative proportion of people surviving over time. [1] [2] This is sometimes presented overall, but frequently within groups, such as randomised arms of a clinical trial. For a clear and simple description of how the Kaplan–Meier estimate is calculated, see Bland and Altman. [3] In producing even a simple Kaplan–Meier plot, there are many choices to be made, leading to wide variation in presentation quality.

Figure 1 gives one example of a Kaplan–Meier plot (based on data from the RT01 trial) [4]. Box 1 outlines the basic anatomy of a Kaplan–Meier plot and highlights some of the choices to be made for readers who are unfamiliar.

Box 1: Anatomy of a Kaplan–Meier plot

In figure 1, the vertical axis runs from 0 to 1 and the horizontal from 0 to 12 years post-randomisation (though this was not the longest follow-up available). The Kaplan–Meier estimate for the control arm is depicted by a red dashed line and for the research arm by a solid blue line. The 'curves' are stepped over time because the estimate changes only at times when an event has occurred. These steps become more pronounced over time as more participants are censored. Beneath the horizontal axis is a table that reports the number of participants still 'at-risk' at specific time points (here 0, 2, 4, 6, 8, 10 and 12 years) *i.e.* they are still in follow-up at this time point, not having had an event or been censored. In figure 1, after 10 years, there remain 71 and 99 participants at risk of an event in the control and research arms respectively.

The utility of a Kaplan–Meier plot depends on who is using it and their purpose. Potential users may be: members of data monitoring committees considering interim data; systematic reviewers extracting data for meta-analysis; trial designers looking for information from relevant patients for sample size calculations; clinicians trying to understand and communicate survival to their patients; and those interested in the value of an estimand that was not reported, such as restricted mean survival times. Even when produced with care, key information may still be lacking for certain readers. It may seem that Kaplan–Meier plots do not require much 'learning to read'. However, we have many times been asked by collaborators how to read them.

We can learn a lot from a Kaplan–Meier plot: the estimated survival fraction at various times; the difference in survival fractions between two groups; quantiles of survival time; suggestions that hazard functions may be non-proportional. [5] It is even possible to reconstitute (data similar to) the underlying survival data based on Kaplan–Meier plots, often very accurately. [6]

Despite the above strengths, there are many issues which may hinder interpretation of Kaplan–Meier plots. The two key factors, and the focus of this work, are to communicate clearly:

1. the number of participants at-risk, censored, and having experienced an event at specific times, over time;
2. the uncertainty of the Kaplan–Meier estimate over time.

These aspects could aid interpretation and increase the amount of information conveyed by Kaplan–Meier plots. We believe a central problem is that a standard Kaplan-Meier plot does not clearly show that the right-hand portion of the curve (at later time points when there are usually considerably fewer

For peer review only - http://bmjopen.bmj.com/site/about/guidelines.xhtml

patients at risk) is estimated with much greater uncertainty than the left-hand portion of the curve. As a consequence, we are concerned that many consumers of Kaplan-Meier curves place undue emphasis on differences between curves at these later time points when differences are much more likely due to chance.

As a snapshot describing recent practice, we reviewed the Kaplan–Meier plots presented in articles published in the BMJ, JAMA, The Lancet and NEJM during 2013. In total, there were 50 randomised, superiority trials with a time-to-event primary outcome. The two dominant specialities were cardiovascular disease (22 trials, 44%) and cancer (11 trials, 22%). Forty seven plots (94%) included a table of the numbers at risk over time, 10 (20%) depicted censoring in some way, either within a table beneath the plot or as ticks on the lines, and five (10%) depicted uncertainty using some form of confidence interval.

The objectives of this work are first, to identify alternatives in relation to the above issues, and second, to understand which (if any) alternatives offer improvements to standard practice.

Methods

Resulting directly from the objectives, the two activities undertaken were:

1. To propose some improvements in Kaplan–Meier plots; and
2. To survey stakeholders in order to understand which are preferred.

Graph development

The constraint on activity (1) was that any proposals should still principally contain a figure showing the Kaplan–Meier estimate over time and should not be based on a different visual description of survival data (such as those in [7] and [8]).

A number of proposals were conceptualised, created and triaged. These were taken forward on the basis of being reasonably different to one another and favoured by at least one of the authors. This resulted in six proposals to take to survey, including four alternative means of representing the numbers at risk and two means of representing uncertainty.

Sources of data and randomisation

With the aim of covering a range of scenarios, we created the proposals for three published, phase III randomised trials:

1. *RT01*: a two-arm trial in prostate cancer which showed a clear difference in biochemical progression-free survival; [4]
2. *ICON7*: a two-arm trial in ovarian cancer with crossing survival curves; [9]
3. *LY09* [9]: a three-arm trial in Hodgkin's lymphoma with limited differences between the arms. [10]

Participants were invited to take a short survey of 13 questions relating to these proposals.

To avoid the repetition and burden of answering all questions for each of the three trials, participants were randomly assigned to see graphs for just one of the trials, using simple randomisation in a 1:1:1

For peer review only

ratio (via a JavaScript tool invoked when a participant clicked the link to take the survey). The purpose of this randomisation was not to compare the randomised groups (as in a randomised trial) but to elicit opinions averaged over these three scenarios.

Survey overview

The survey asked for participants' opinions and preferences regarding the six alternatives as compared with a reference that we regard as a reasonably 'standard' Kaplan–Meier plot (similar tofigure 1; note that what constitutes 'standard' is subject to opinion). The proposals are shown in figure 2 (for the RT01 trial data, with 'standard' based on authors' consensus). Larger versions of each graph are given in the supplementary file (supplementary figures 1–18) for all three trials.

In order to understand which of these proposals were preferred by stakeholders, we conducted a survey using Online Surveys (formerly BOS) [11].

Taking the survey

Participants were shown each proposed graph and asked to score it on a five-point ordinal scale, against the reference graph (without the proposed alteration, similar to that in figure 1), with the reference and proposal options visible side-by-side. The options were 'Less useful', 'Equal/no preference', 'A bit more useful', 'Somewhat more useful' and 'Much more useful'. Participants were next asked to rank (in order) up to three preferred proposals and to not rank any proposal that they disliked. This ranking was done separately, once for the proposals addressing the numbers-at-risk and once for those addressing uncertainty.

After answering each of the above questions, participants had an opportunity to provide free-text comments, and a further opportunity to provide general comments on the survey. This gave a chance to explain their ratings of graphs. All of the free-text comments were read and categorised by the authors, with participants' comments assigned completely at random to one of BCO, CIJ, MJS, TPM or WJC. These comments were categorised in two ways. First, many of the comments were categorised as being to criticise, praise or suggest improvements to one of the proposals (most proposal-specific comments fell into one of these categories). Secondly, we categorised further comments (not proposal-specific) according to the comments made.

Baseline information collected

As well as opinions, we collected some participant characteristics. For descriptive purposes, we collected the country in which participants are primarily based, the date on which the survey was taken, and the years of experience 1) 'reading and interpreting', and 2) 'creating' Kaplan–Meier graphs. To explore whether opinions varied according to two specific characteristics, we also asked what participants identified as their principal professional background and whether or not they currently act as a journal editor. We regard the latter as important because journals often specify styles for Kaplan–Meier plots (either in instructions to authors or during typesetting) and so editors may exert disproportionate influence over what appears in the literature.

Recruiting participants

We recruited participants by publicising the survey through many channels: emails to colleagues and collaborators, Twitter, email lists including AllStat and the ISCB list, clinical collaborators of the MRC

For peer review only

Clinical Trials Unit at UCL, the UK Hubs for Trials Methodology Research (note that this list is non-exhaustive). As the survey ran, we noted the high proportion of participants whose primary role was statistician, and so targeted clinicians and systematic reviewers more purposefully.

Analysis of results

Analysis of the data is descriptive, generally depicting the frequency of specific responses in graphs. The data on which the analysis is based are provided in the supplementary file for readers to explore themselves, minus the date of survey, free-text comments and participant country.

Note on 'sampling'

The survey did not have any formal sampling mechanism (or well-defined units of the population, or its size) and is a convenience sample. We targeted those that we view as users and/or creators of Kaplan–Meier plots and who we could reach, for example, registered clinical trials units in the UK, journal editors, and systematic reviewers.

Data availability

De-identified data containing individual responses to the survey will be made openly available on publication. De-identification necessarily required removal of some of the descriptive variables, including free-text comments (some comments made participants identifiable) and country (continent is retained).

Ethics approval

No ethical approval was required (or obtained), assessed using the online HRA decision tool. This was a survey of opinions on a non-sensitive subject, collecting no biological samples or data that might be identifiable (unless participants identified themselves in a free-text comment or chose to provide contact details to hear about the results of the survey).

Results

One thousand, two hundred and seventy four participants completed the survey between 26 Apr 2017 and 7 July 2017.

Figure 3 gives descriptive information about the participants: self-described primary role/training, country in which they primarily work, whether they act as a journal editor, and experience (i) reading/interpreting and (ii) producing Kaplan–Meier plots. Supplementary figure 19 is a plot of experience *reading/interpreting vs. producing* Kaplan–Meier plots.

The most represented roles were statistician (727; 57%) and clinician (341; 27%). Several other groups were well represented (see figure 3), but the results will be dominated by the groups identifying themselves as statistician or clinician. One hundred and seventy (14%) respondents identified themselves as journal editors. Participants were based primarily in the UK and USA but 36% were based in other countries, representing all populated continents (see figure 3).

For peer review only

Participants' opinions on the proposed alterations to Kaplan–Meier plots are given in figure 4. The upper row of figure 4a contains the proposals for presenting how the number at risk changes over time; the lower row of figure 4a for those depicting uncertainty. On the upper row, the extended risk-table garnered the most positive opinions, with 1,054 (83%) participants giving a positive response. Some (110 participants; 9%) found this extra information 'less useful'. Using a line graph to depict the numbers at risk was not popular, with 574 (45%) finding this less useful than the usual table depicting numbers at risk. The graph of areas to replace the extended risk-table divided opinion: while 347 (27%) found it less useful than a standard plot, 772 (61%) found it 'a bit', 'somewhat' or 'much more' useful. The same chart with the areas superimposed behind the Kaplan–Meier estimate was much less popular, with 720 (57%) finding it less useful than the usual plot (at this first exposure). The lower row shows ambivalence about the idea of faded lines: 545 (43%) found this less useful than a standard presentation, 195 (15%) had no preference and 534 (42%) found it more useful.

These results were broadly similar across the three trials, both for statisticians and clinicians, and for editors and non-editors of journals. Figures similar to the upper panel of figure 4, broken down by these groups, can be found in the supplementary file.

Figure 4b gives participants' overall rankings for the proposals, separately for those addressing numbers at risk and uncertainty. Green bars depict the number of participants who ranked this graph as their first choice; orange as second, red as third, and grey not ranked (for proposals depicting uncertainty, although there were only three options, participants did not have to answer for all choices if, for example, they found only one option to be acceptable). These results give the same message as those presented in figure 4a. For presenting numbers at risk, an extended risk table is the clear favourite; for depicting uncertainty, use of confidence intervals was the first choice for over half the participants.

An idea of the nature of free text responses is provided in supplementary figure 20, which summarises whether graph-specific comments were criticism, praise, or suggestions (left) and gives the broad types of comment (right).

Discussion

We have proposed several alterations to 'standard' Kaplan–Meier plots, specifically for the context of showing within-arm survival in randomised trials. The proposals were around two key aspects depicting: 1) the numbers at risk over time and 2) uncertainty.

We then surveyed users of Kaplan–Meier plots for their views on our proposals. Several garnered more positive opinions than the reference plot, and two came out as the overall favourites, although opinions were far from unanimous.

We do not make explicit recommendations here about which alterations should be used but encourage producers of Kaplan–Meier plots and those who influence them (journal editors and regulators) to consider their practice in light of these results. In particular, the plots including an extended table of numbers and confidence intervals seemed to be favoured by most participants. These can be used in combination without any clash, and we include an example with both aspects in figure 5, again using the RT01 data.

For peer review only

There is clear recognition that graphical representations of time-to-event data could be improved. Many free text responses noted context. Kaplan–Meier plots are used by: trial designers looking for previous information on a related group of patients; data monitoring committees viewing interim data; meta-analysts to extract data; and clinicians looking to understand and communicate risks to their patients. There is no one-size that fits all settings and producers of Kaplan–Meier plots need to make judicious choices according to their context.

The two proposals involving area graphs to depict the number at risk require some thought to understand and are not instantly readable; a graph which requires little 'learning to read' is perhaps desirable. These two proposals were broadly unpopular in the survey: Many commented that this depiction was confusing, but a minority who liked them said it took time to reach that conclusion. Prior to the survey, the authors had expected the KM curve superimposed on the area depicting numbers at risk to be more popular than they were. The results of the survey show the desirability of a graph that requires little 'learning to read' and also the importance of a large stakeholder survey to elicit representative preferences.

Depicting the numbers-at-risk using line charts below the Kaplan–Meier plot was also reasonably unpopular. Free text comments suggested three main reasons: 1) participants wanted specific numbers in preference to a general pattern; 2) the line looks similar to the line of the Kaplan–Meier estimate, leading to potential confusion; and 3) as we created and presented this option, the plot region for the numbers in follow-up used 1/3 the area of the plot region for the Kaplan–Meier estimate, which for some participants was inadequate – a poor choice on our part. This proportion would need to be reconsidered by anyone looking to use the approach.

For depicting uncertainty, fading the Kaplan–Meier estimates was unpopular. There were two principal reasons for this. Firstly, when printed, the fading could be confused for a printing error, rather than an intended effect. Secondly, it is not clear how to define the level of decreasing intensity that accurate reflects the readers' perception of increasing uncertainty. A minor comment from some clinicians was the desire to be able to accurately read the estimate at a very late time point (note that the premise for use of fading was in part to prevent this where uncertainty is extremely high).

Further thought is required on visualising survival data, and new proposals would ideally be accompanied by studies on stakeholders' opinions. We constrained this project to Kaplan–Meier plots with two or more groups. However, in a randomised trial we are interested in comparing arms and so want to visualise some estimate of the difference. Such visualisations may be a fruitful future direction.

Interestingly, Paul Meier himself is said to have spoken with bemusement about people plotting Kaplan–Meier estimates over time and was not convinced he actually liked it (the authors thank Chris Barker, a former student of Meier, for this personal communication).

As noted in the methods section, the trial datasets we used do not represent any true distribution of scenarios occurring in clinical trials. Rather, they represent a small variety of situations which can occur in randomised trials; for any change to Kaplan–Meier plots to be worthwhile, the impact of features such as non-inferiority, non-proportional hazards, more than two arms, and different allocation ratios should be assessed. Having said this, if a plot works well for two-arm trials but not three-arm trials, it may of course be used in that context.

For peer review only

We hope that this work will provoke those creating Kaplan–Meier plots to think carefully about how they can best convey the information, and that journal editors will consider their policies for rendering Kaplan–Meier plots. We will continue to consider alternatives and evaluate these in the future.

Acknowledgements

We are grateful to the 1,274 participants who provided their opinions, particularly those who pointed us to articles containing their own thoughts on Kaplan–Meier; to the ICON7, LY09 and RE01 trial teams and participants for the use of these data.

Funding

This work was supported by the Medical Research Council (grant numbers MC_UU_12023/21, MC_UU_12023/29 and MC_UU_12023/24) – TM, WC, PP, BCO, MS.

Conflicts of Interest

The authors declare that they have no relevant conflicts of interest.

References

[1] E. L. Kaplan and P. Meier, "Nonparametric Estimation from Incomplete Observations," *Journal of the American Statistical Association,* vol. 53, pp. 457-481, 1958.

[2] S. J. Pocock, T. G. Travison and L. M. Wruck, "Figures in clinical trial reports: current practice & scope for improvement," *Trials,* vol. 8, p. 36+, 2007.

[3] J. M. Bland and D. G. Altman, "Survival probabilities (the Kaplan-Meier method)," *BMJ,* vol. 317, pp. 1572-1580, 1998.

[4] D. P. Dearnaley, G. Jovic, I. Syndikus, V. Khoo, R. A. Cowan, J. D. Graham, E. G. Aird, D. Bottomley, R. A. Huddart, C. C. Jose, J. H. L. Matthews, J. L. Millar, C. Murphy, J. M. Russell, C. D. Scrase, M. K. B. Parmar and M. R. Sydes, "Escalated-dose versus control-dose conformal radiotherapy for prostate cancer: long-term results from the MRC RT01 randomised controlled trial," *The Lancet Oncology,* vol. 15, pp. 464-473, 2014.

[5] I. Ford and J. Norrie, "The role of covariates in estimating treatment effects and risk in long-term clinical trials," *Statist. Med.,* vol. 21, pp. 2899-2908, 15 10 2002.

[6] P. Guyot, A. E. Ades, M. J. Ouwens and N. J. Welton, "Enhanced secondary analysis of survival data: reconstructing the data from published Kaplan-Meier survival curves," *BMC Medical Research Methodology,* vol. 12, p. 9+, 2012.

[7] J. Newell, J. W. Kay and T. C. Aitchison, "Survival ratio plots with permutation envelopes in survival data problems," *Computers in Biology and Medicine,* vol. 36, pp. 526-541, 2006.

[8] P. Royston, M. K. B. Parmar and D. G. Altman, "Visualizing Length of Survival in Time-to-Event Studies: A Complement to Kaplan Meier Plots," *JNCI Journal of the National Cancer Institute,* vol. 100, pp. 92-97, 2008.

[9] A. M. Oza, A. D. Cook, J. Pfisterer, A. Embleton, J. A. Ledermann, E. Pujade-Lauraine, G.

For peer review only

Kristensen, M. S. Carey, P. Beale, A. Cervantes, T. W. Park-Simon, G. Rustin, F. Joly, M. R. Mirza, M. Plante, M. Quinn, A. Poveda, G. C. Jayson, D. Stark, A. M. Swart, L. Farrelly, R. Kaplan, M. K. B. Parmar and T. J. Perren, “Standard chemotherapy with or without bevacizumab for women with newly diagnosed ovarian cancer (ICON7): overall survival results of a phase 3 randomised trial,” *The Lancet Oncology,* vol. 16, pp. 928-936, 2015.

[10] P. W. M. Johnson, M. R. Sydes, B. W. Hancock, M. Cullen, J. A. Radford and S. P. Stenning, “Consolidation Radiotherapy in Patients With Advanced Hodgkin's Lymphoma: Survival Data From the UKLG LY09 Randomized Controlled Trial (ISRCTN97144519),” *Journal of Clinical Oncology,* vol. 28, pp. 3352-3359, 2010.

[11] *Online Surveys (formerly BOS),* 2019.

Captions for figures

Figure 1. An example of a Kaplan–Meier plot from the RT01 trial

Figure 2. Proposed graphs using the RT01 data. A) An extended table showing the status of participants over time; B) A plot of the number of participants at-risk over time by arm; C) A plot of the status of participants over time by arm, beneath the Kaplan–Meier plot; D) Two plots of the status of participants over time, one for each arm, behind the Kaplan–Meier plot; E) Confidence intervals presented around the Kaplan–Meier estimate; D) Fading of the Kaplan–Meier lines as information reduces.

Figure 3. Descriptive characteristics of participants (n=1,274) as a dot chart (% on horizontal axis; frequencies labelled directly)

Figure 4. A) Opinion of alteration vs. `standard' Kaplan–Meier plot. Upper row is for alterations in presenting numbers at risk; lower row is for alterations in depicting uncertainty. B) Participants' overall preferences for presenting numbers at risk (upper part) and depicting uncertainty (lower part)

Figure 5. The two most popular elements combined: confidence intervals and extended at-risk table

For peer review only

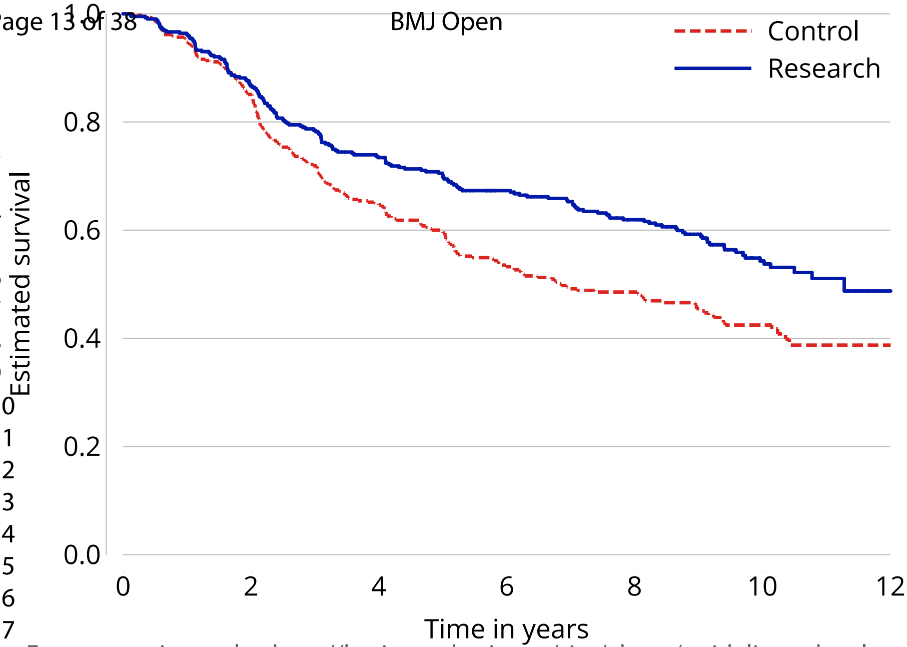

At risk

	0	2	4	6	8	10	12
Control	418	351	255	190	154	71	6
Research	418	351	283	239	198	99	8

For peer review only - http://bmjopen.bmj.com/site/about/guidelines.xhtml

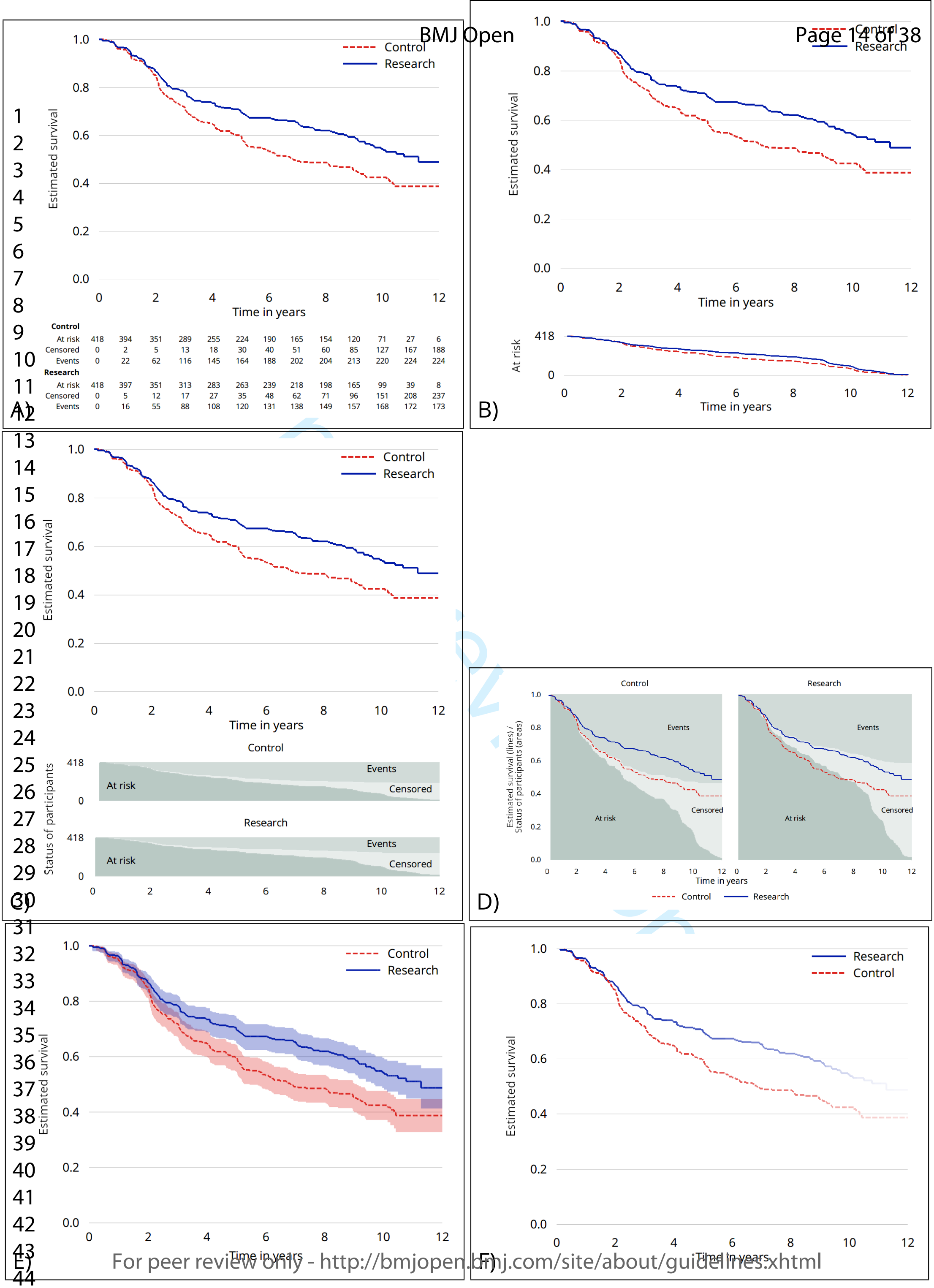
A)
Estimated survival
1.0
0.8
0.6
0.4
0.2
0.0
0 2 4 6 8 10 12
Time in years
Control
Research
Control
At risk 418 394 351 289 255 224 190 165 154 120 71 27 6
Censored 0 2 5 13 18 30 40 51 60 85 127 167 188
Events 0 22 62 116 145 164 188 202 204 213 220 224 224
Research
At risk 418 397 351 313 283 263 239 218 198 165 99 39 8
Censored 0 5 12 17 27 35 48 62 71 96 151 208 237
Events 0 16 55 88 108 120 131 138 149 157 168 172 173
B)
Estimated survival
Time in years
Control
Research
At risk
418
0
C)
Estimated survival
Time in years
Control
Research
Status of participants
Control
Research
418
0
At risk
Events
Censored
D)
Control
Research
Estimated survival (lines) / Status of participants (areas)
Events
Censored
At risk
Time in years
Control
Research
E)
Estimated survival
Time in years
Control
Research
F)
Estimated survival
Time in years
Research
Control

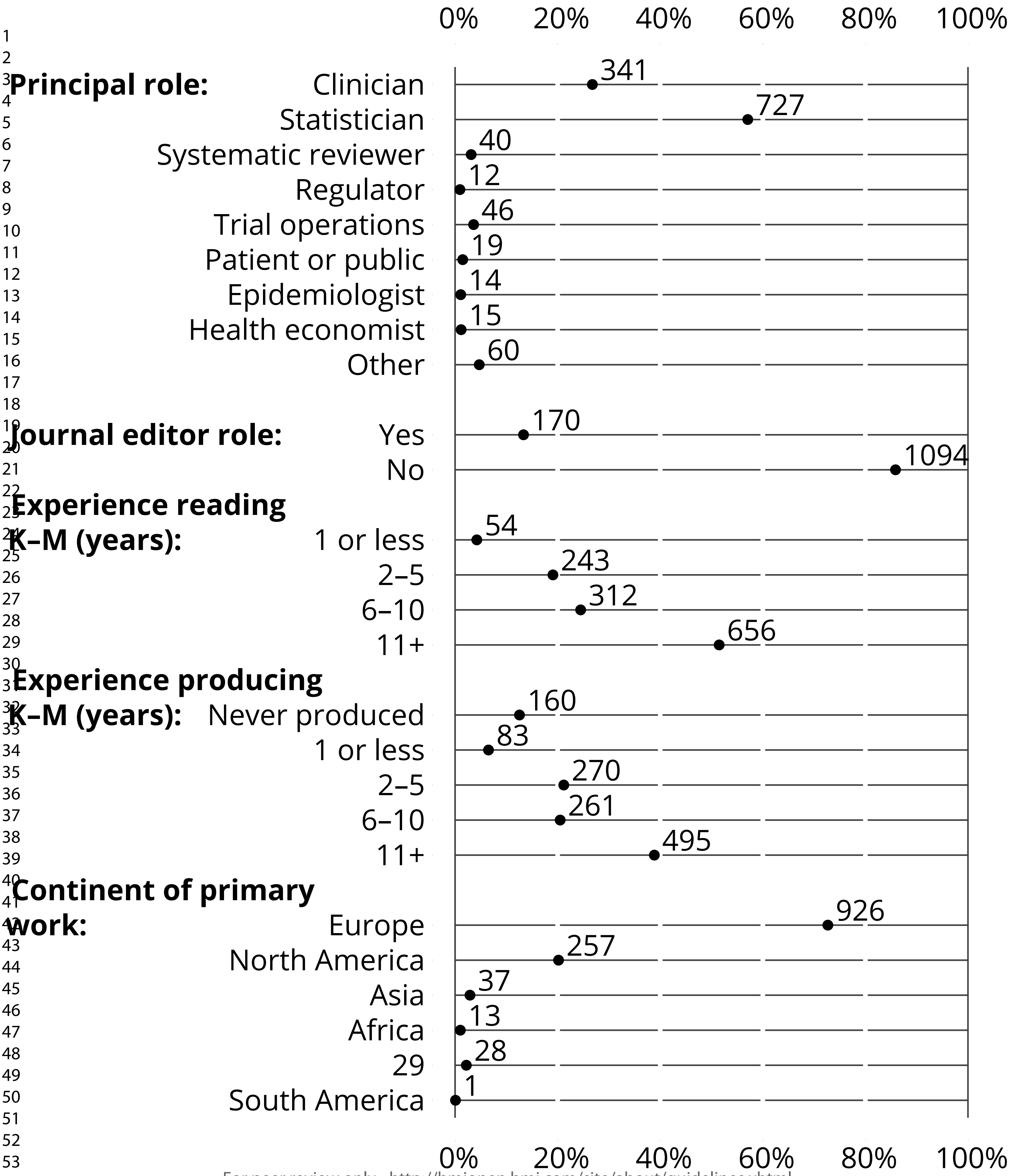
0%
20%
40%
60%
80%
100%
Principal role:
Clinician
341
Statistician
727
Systematic reviewer
40
Regulator
12
Trial operations
46
Patient or public
19
Epidemiologist
14
Health economist
15
Other
60
Journal editor role:
Yes
170
No
1094
Experience reading K–M (years):
1 or less
54
2–5
243
6–10
312
11+
656
Experience producing K–M (years):
Never produced
160
1 or less
83
2–5
270
6–10
261
11+
495
Continent of primary work:
Europe
926
North America
257
Asia
37
Africa
13
29
28
South America
1

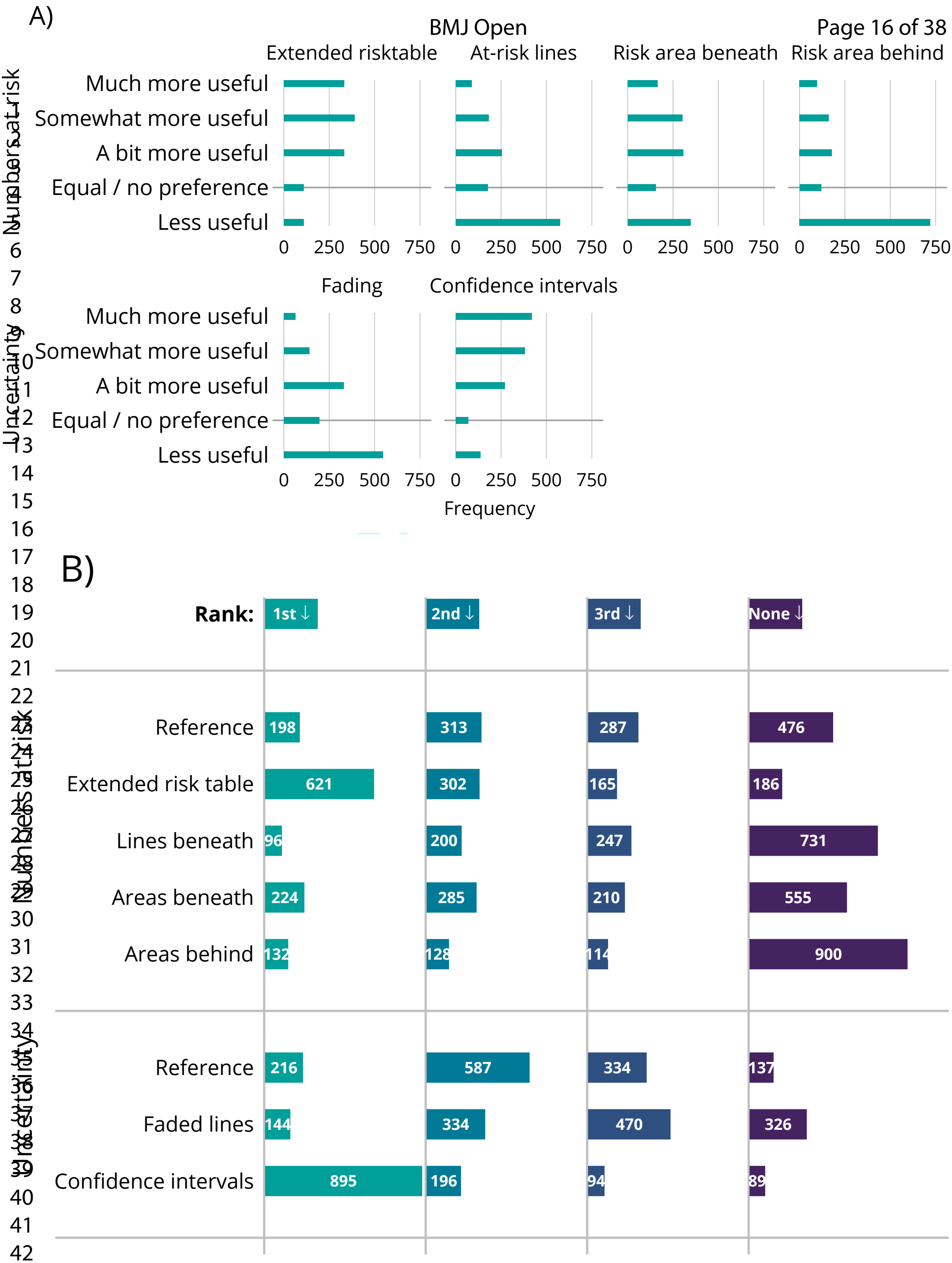
A)
Numbers at risk
Extended risktable
At-risk lines
Risk area beneath
Risk area behind
Much more useful
Somewhat more useful
A bit more useful
Equal / no preference
Less useful
0
250
500
750
Uncertainty
Fading
Confidence intervals
Frequency
B)
Rank:
1st ↓
2nd ↓
3rd ↓
None ↓
Numbers at risk
Reference 198 313 287 476
Extended risk table 621 302 165 186
Lines beneath 96 200 247 731
Areas beneath 224 285 210 555
Areas behind 132 128 114 900
Uncertainty
Reference 216 587 334 137
Faded lines 144 334 470 326
Confidence intervals 895 196 94 89

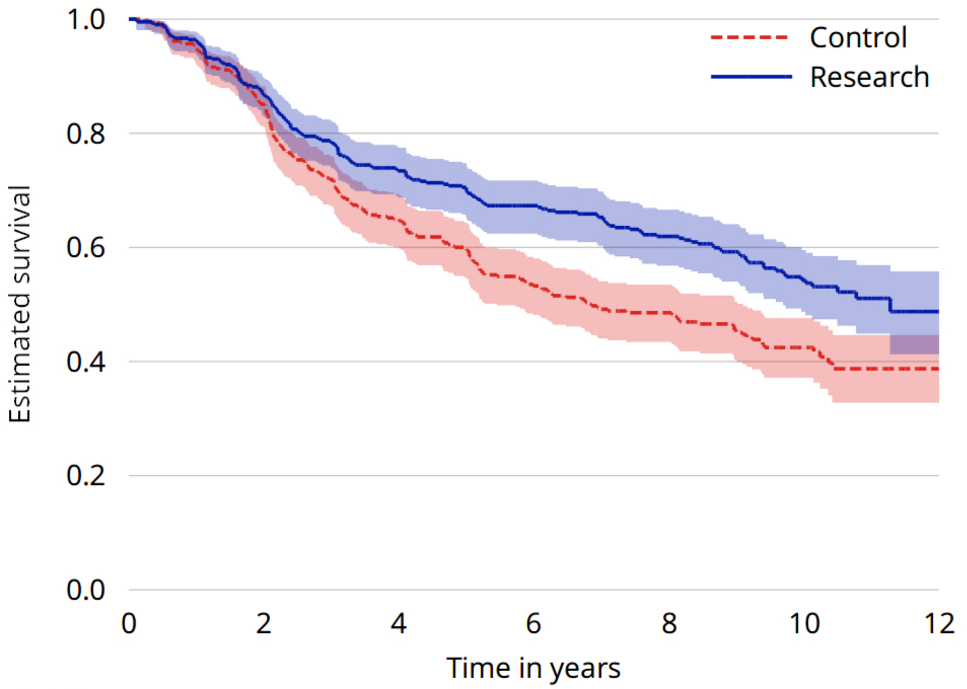

**Control**													
At risk	418	394	351	289	255	224	190	165	154	120	71	27	6
Censored	0	2	5	13	18	30	40	51	60	85	127	167	188
Events	0	22	62	116	145	164	188	202	204	213	220	224	224
**Research**													
At risk	418	397	351	313	283	263	239	218	198	165	99	39	8
Censored	0	5	12	17	27	35	48	62	71	96	151	208	237
Events	0	16	55	88	108	120	131	138	149	157	168	172	173

Figure 5. The two most popular elements combined: confidence intervals and extended at-risk table

1 Supplementary information

1.1 Free text comments on survey (figure 19)

1.2 Email invitation text

Dear *name*

Problem
The standard way to present time-to-event data, such as survival, is with Kaplan–Meier plots. These are formatted by journals and reported in a number of ways, but we find they frequently lack some key information.
The key problems are:

1. Expressing how many people are contributing data at any point in the graph, including the pattern of censoring

2. Expressing that the uncertainty of the estimate increases over time

Suggestion
We have some initial suggestions on how to improve Kaplan–Meier plots, but we need your help to know which would be the most useful and most acceptable to a wide audience.

Invitation to a short survey
Could you take our short survey of nine meaningful multiple choice questions?

You will be asked to compare standard and alternative graphs, using data from one of three RCTS, chosen at random when you follow this link: bit.ly/KMunicate or http://www.ctu.mrc.ac.uk/resources/Kaplan-Meier/index.html.

Please complete the survey in one attempt as we cannot guarantee you will return to the same trial.

The survey will be open until **09-Jun-2017**.

Survey results
The findings will be written up for publication in a peer-reviewed journal and also introduced in an active poster session at the joint SCT & ICTMC 2017 conference in Liverpool.

We are interested to hear from anyone who looks at survival curves and are casting our net as wide as possible. Please forgive us if you have already received an invitation through another means.

If you have colleagues you think would be interested (including clinicians, journal editors, operations specialists, systematic reviewers, regulators, statisticians and trialists), please feel free to forward our invitation and link.

Thank you for your time.

Project team
Tim Morris, Chris Jarvis, Will Cragg, Babak Oskooei, Patrick Philips and Matt Sydes.

For peer review only - http://bmjopen.bmj.com/site/about/guidelines.xhtml

1.3 Proposed alterations as presented to survey participants

The following supplementary figures are provided for readers to see the options we presented to survey participants and the descriptions from the survey. Figures 1, 2, 3, 4, 5 and 6 used data from the *RT01* trial; figures 7, 8, 9, 10, 11 and 12 used data from the *ICON7* trial; figures 13, 14, 15, 16, 17 and 18 used data from the *LY09* trial.

Figure 1: The extended at-risk table (*RT01* trial). The usual table beneath the plot contains the cumulative numbers censored by time t and the cumulative number of events. Note that, at any time point, the three numbers sum to the number at risk at time 0.

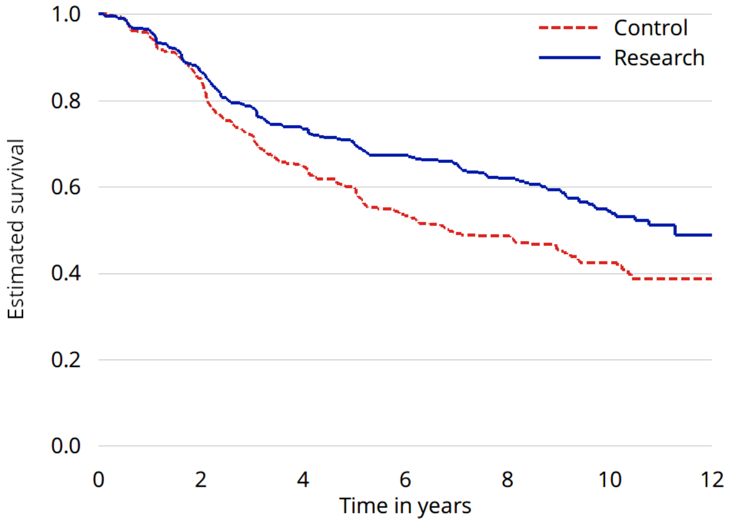

**Control**													
At risk	418	394	351	289	255	224	190	165	154	120	71	27	6
Censored	0	2	5	13	18	30	40	51	60	85	127	167	188
Events	0	22	62	116	145	164	188	202	204	213	220	224	224
**Research**													
At risk	418	397	351	313	283	263	239	218	198	165	99	39	8
Censored	0	5	12	17	27	35	48	62	71	96	151	208	237
Events	0	16	55	88	108	120	131	138	149	157	168	172	173

only

For peer review only - http://bmjopen.bmj.com/site/about/guidelines.xhtml

Figure 2: At-risk lines (*RT01* trial). The usual table of numbers at risk is replaced by a line graph of the numbers at risk over time. It is effectively a less granular version but does not display the exact numbers at risk.

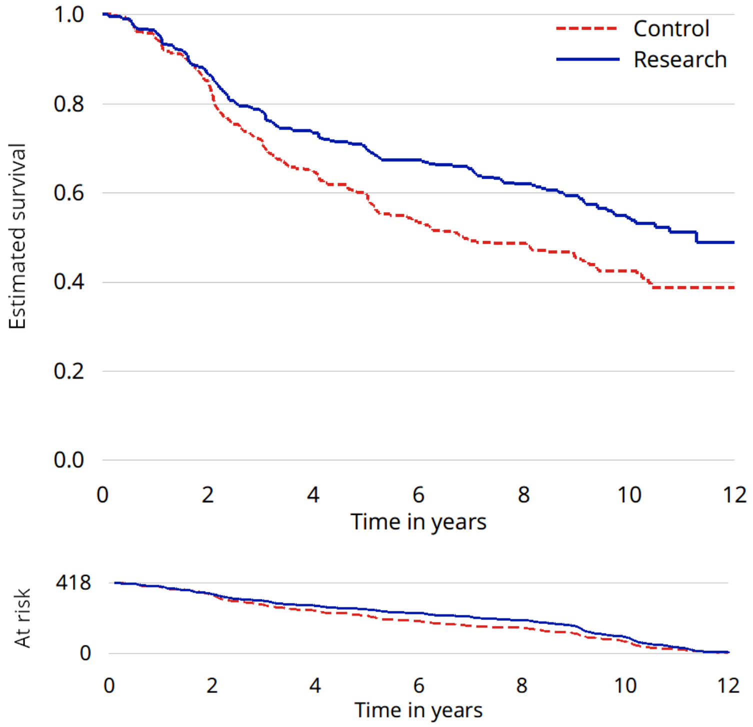

review only

For peer review only - http://bmjopen.bmj.com/site/about/guidelines.xhtml

Figure 3: At-risk areas beneath (*RT01* trial). This is a graphical form of the extended at-risk table. By arm, the cumulative number at risk, censored, and experiencing an event are given beneath the Kaplan–Meier plot.

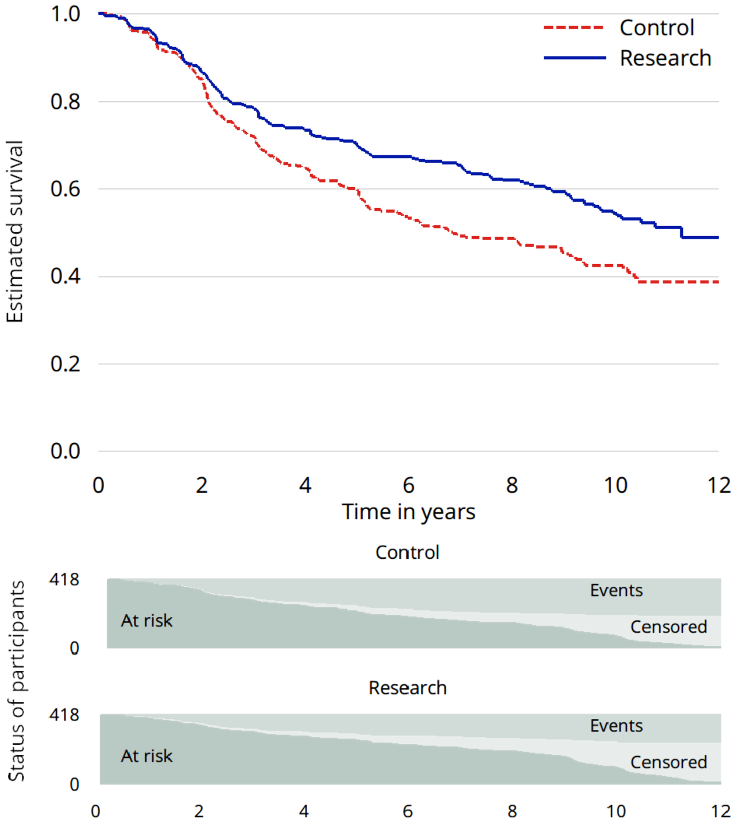

For peer review only - http://bmjopen.bmj.com/site/about/guidelines.xhtml

Figure 4: At-risk areas behind the Kaplan–Meier plot (*RT01* trial). The graphical at-risk graphs are now drawn behind the Kaplan–Meier plot. Because there is one area graph for each arm, this necessitates repeating the Kaplan–Meier curves as many times as there are randomised arms.

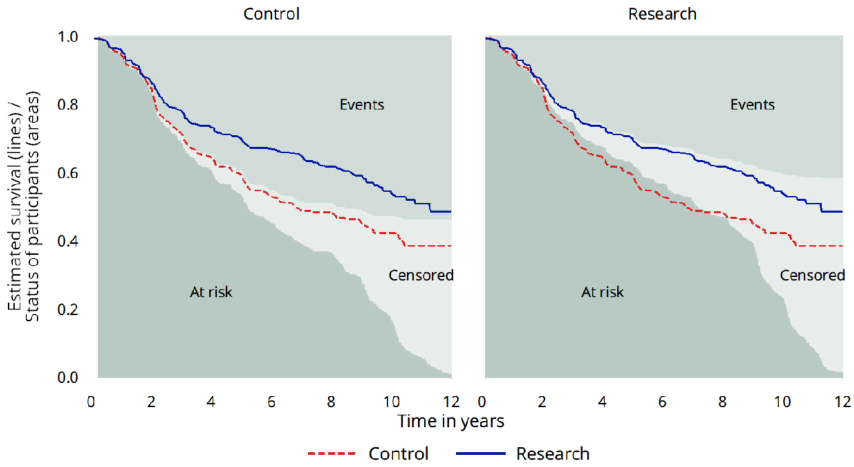

peer review only

For peer review only - http://bmjopen.bmj.com/site/about/guidelines.xhtml

Figure 5: Confidence intervals to depict uncertainty (*RT01* trial). Here, point-wise confidence intervals are plotted around the Kaplan–Meier estimate. We chose to plot these by shading of the area within the interval using the same colour as the line translucent, thus areas of overlap can be clearly seen.

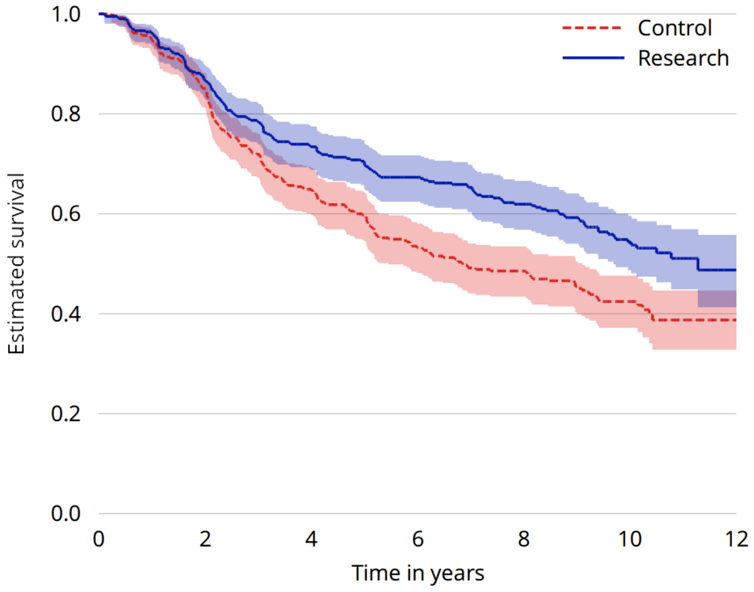

For peer review only

For peer review only - http://bmjopen.bmj.com/site/about/guidelines.xhtml

Figure 6: Fading of the Kaplan–Meier estimates to depict uncertainty (*RT01* trial). Here, the curves fade in proportion to the cumulative number pf censored individuals (since it is censoring, not events, which means the estimate becomes more uncertain as time passes). The aim is to explicitly give the reader a visual deterrent when the eye is drawn to the far right.

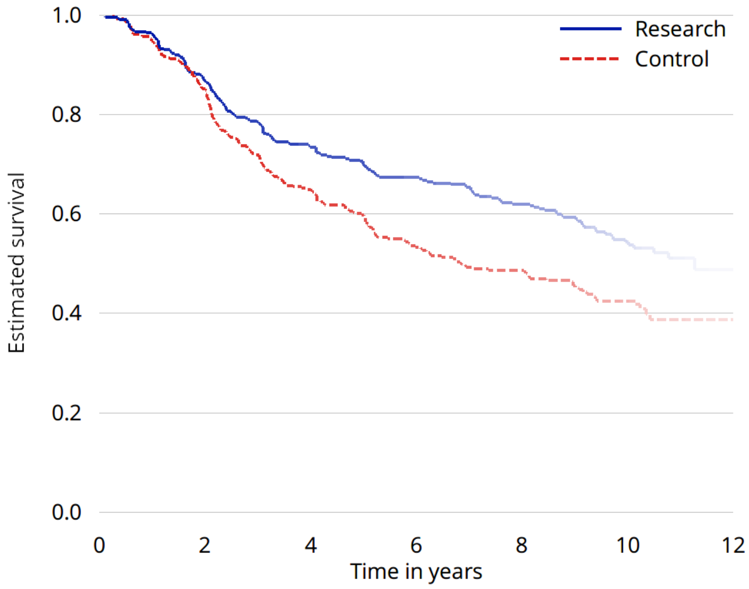

For peer review only - http://bmjopen.bmj.com/site/about/guidelines.xhtml

Figure 7: The extended at-risk table (*ICON7* trial). The usual table beneath the plot contains the cumulative numbers censored by time t and the cumulative number of events. Note that, at any time point, the three numbers sum to the number at risk at time 0.

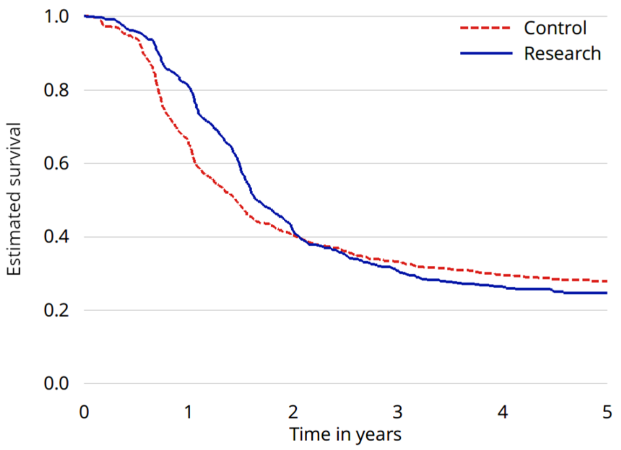

		0	1	2	3	4	5
**Control Arm**	At risk	764	477	293	236	194	61
	Events	0	256	438	490	515	523
	Censored	0	31	33	38	55	180
**Research Arm**	At risk	764	600	306	224	177	49
	Events	0	143	434	514	545	553
	Censored	0	21	24	26	42	162

For peer review only

Figure 8: At-risk lines (*ICON7* trial). The usual table of numbers at risk is replaced by a line graph of the numbers at risk over time. It is effectively a less granular version but does not display the exact numbers at risk.

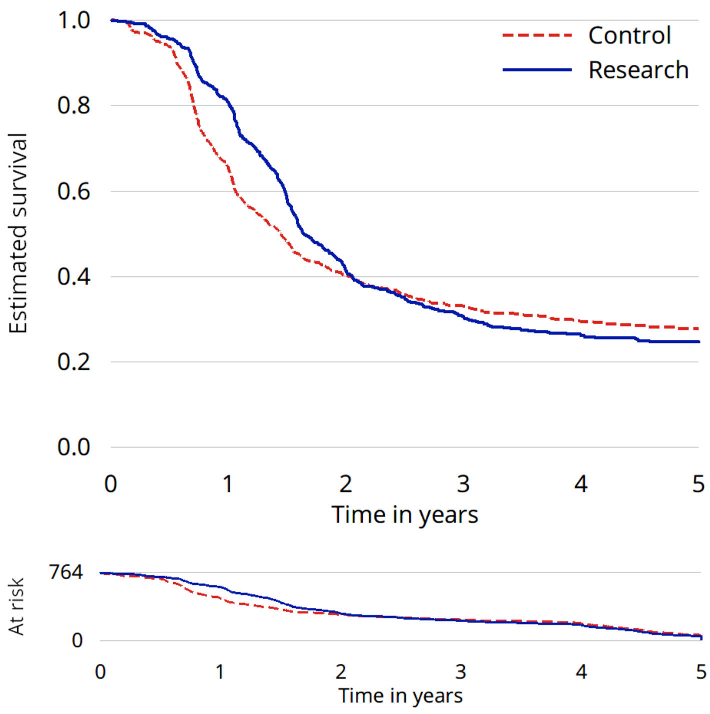

review only

For peer review only - http://bmjopen.bmj.com/site/about/guidelines.xhtml

Figure 9: At-risk areas beneath (*ICON7* trial). This is a graphical form of the extended at-risk table. By arm, the cumulative number at risk, censored, and experiencing an event are given beneath the Kaplan–Meier plot.

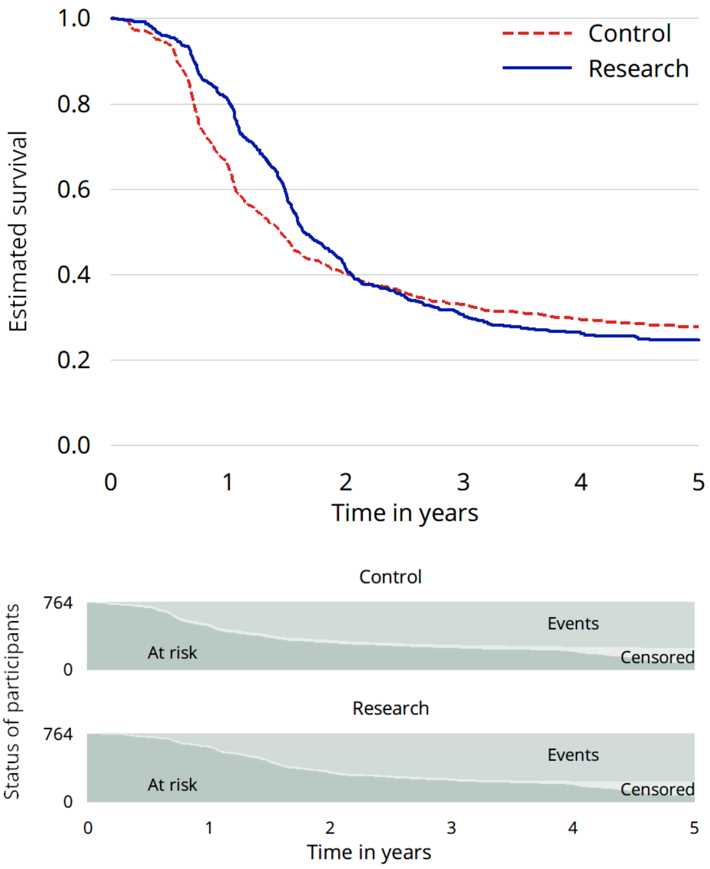

For peer review only

For peer review only - http://bmjopen.bmj.com/site/about/guidelines.xhtml

Figure 10: At-risk areas behind the Kaplan–Meier plot (*ICON7* trial). The graphical at-risk graphs are now drawn behind the Kaplan–Meier plot. Because there is one area graph for each arm, this necessitates repeating the Kaplan–Meier curves as many times as there are randomised arms.

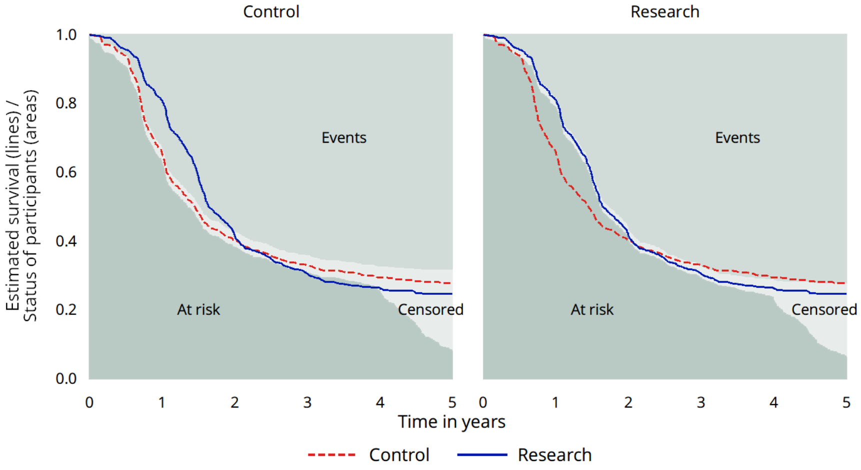

For peer review only

For peer review only - http://bmjopen.bmj.com/site/about/guidelines.xhtml

Figure 11: Confidence intervals to depict uncertainty (*ICON7* trial). Here, point-wise confidence intervals are plotted around the Kaplan–Meier estimate. We chose to plot these by shading of the area within the interval using the same colour as the line translucent, thus areas of overlap can be clearly seen.

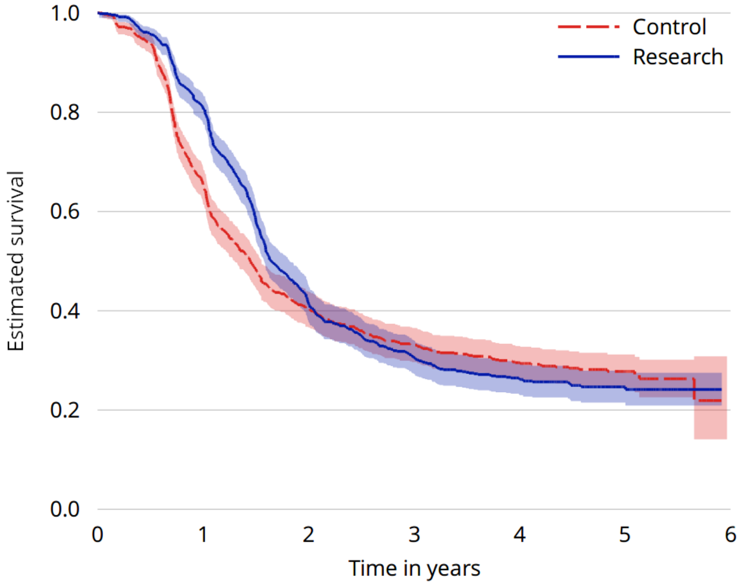

For peer review only

For peer review only - http://bmjopen.bmj.com/site/about/guidelines.xhtml

Figure 12: Fading of the Kaplan–Meier estimates to depict uncertainty (*ICON7* trial). Here, the curves fade in proportion to the cumulative number of censored individuals (since it is censoring, not events, which means the estimate becomes more uncertain as time passes). The aim is to explicitly give the reader a visual deterrent when the eye is drawn to the far right.

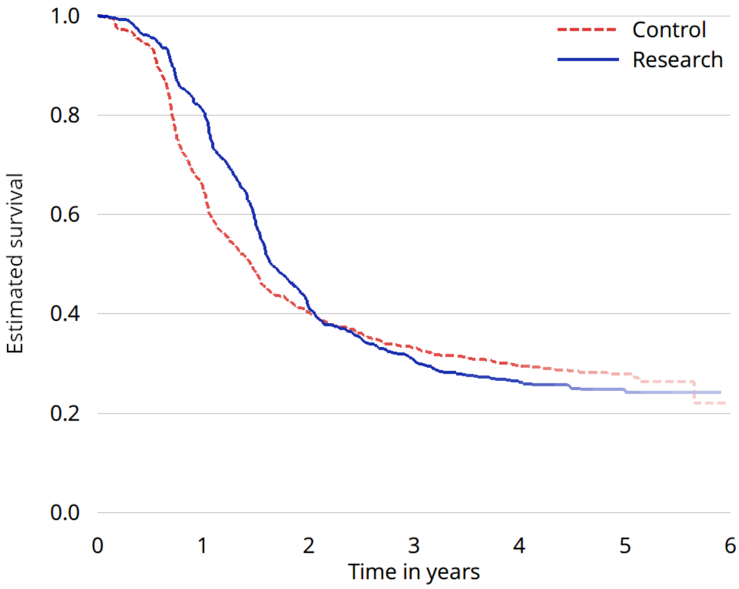

For peer review only

Figure 13: The extended at-risk table (*LY09* trial). The usual table beneath the plot contains the cumulative numbers censored by time t and the cumulative number of events. Note that, at any time point, the three numbers sum to the number at risk at time 0.

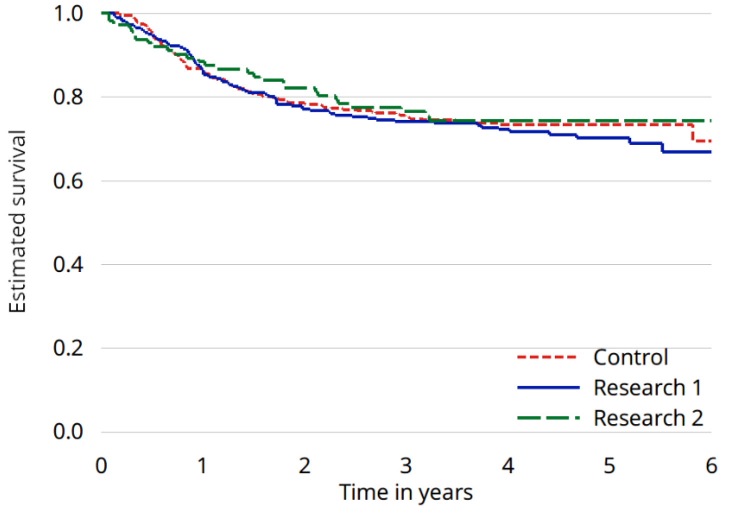

	0	1	2	3	4	5	6
**Control**							
At risk	394	338	302	265	178	79	10
Censored	0	3	7	33	114	213	281
Events	0	53	85	96	102	102	103
**Research 1**							
At risk	282	241	210	182	137	69	8
Censored	0	3	8	28	69	134	193
Events	0	38	64	72	76	79	81
**Research 2**							
At risk	112	99	90	75	52	25	3
Censored	0	0	2	11	32	59	81
Events	0	13	20	26	28	28	28

view only

For peer review only - http://bmjopen.bmj.com/site/about/guidelines.xhtml

Figure 14: At-risk lines (*LY09* trial). The usual table of numbers at risk is replaced by a line graph of the numbers at risk over time. It is effectively a less granular version but does not display the exact numbers at risk.

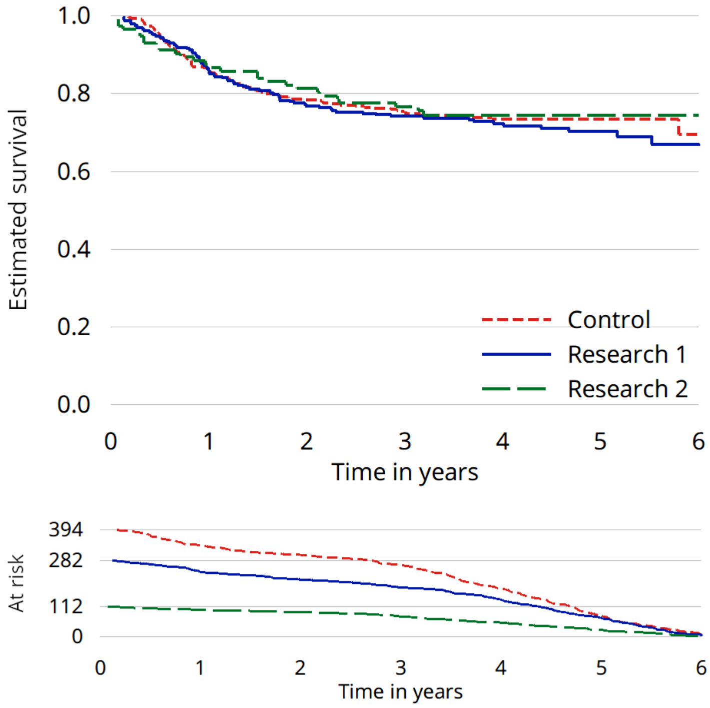

review only

For peer review only - http://bmjopen.bmj.com/site/about/guidelines.xhtml

Figure 15: At-risk areas beneath (*LY09* trial). This is a graphical form of the extended at-risk table. By arm, the cumulative number at risk, censored, and experiencing an event are given beneath the Kaplan–Meier plot.

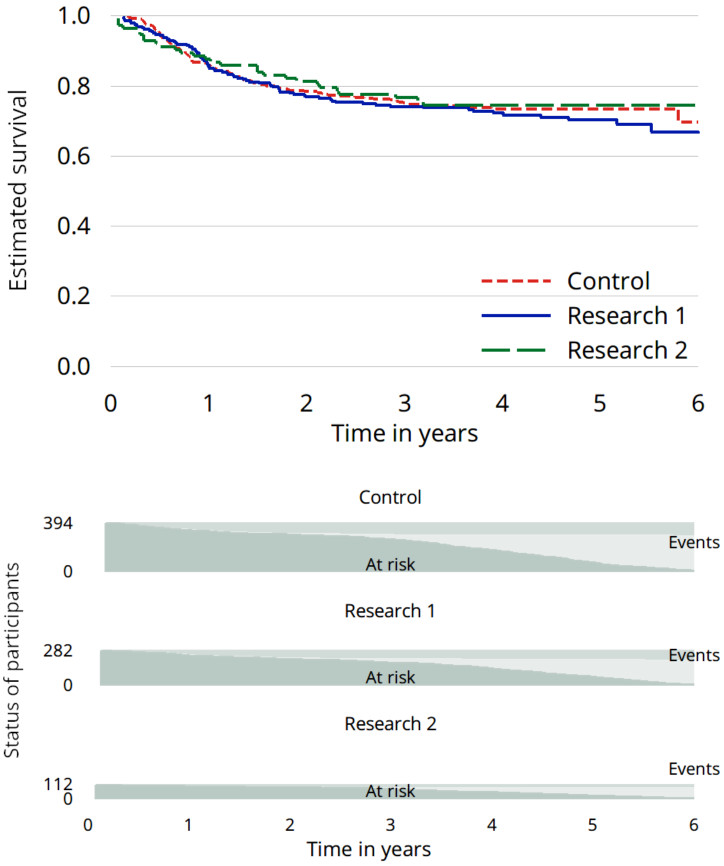

For peer review only - http://bmjopen.bmj.com/site/about/guidelines.xhtml

Figure 16: At-risk areas behind the Kaplan–Meier plot (*LY09* trial). The graphical at-risk graphs are now drawn behind the Kaplan–Meier plot. Because there is one area graph for each arm, this necessitates repeating the Kaplan–Meier curves as many times as there are randomised arms.

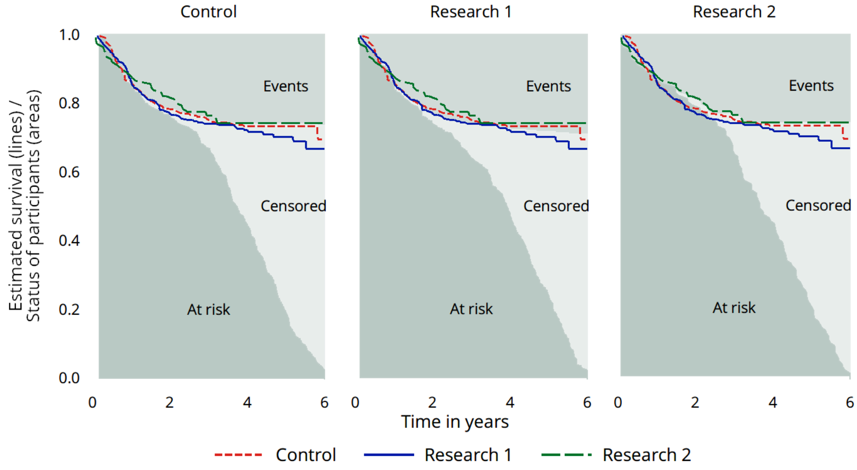

For peer review only

Figure 17: Confidence intervals to depict uncertainty (*LY09* trial). Here, point-wise confidence intervals are plotted around the Kaplan–Meier estimate. We chose to plot these by shading of the area within the interval using the same colour as the line translucent, thus areas of overlap can be clearly seen.

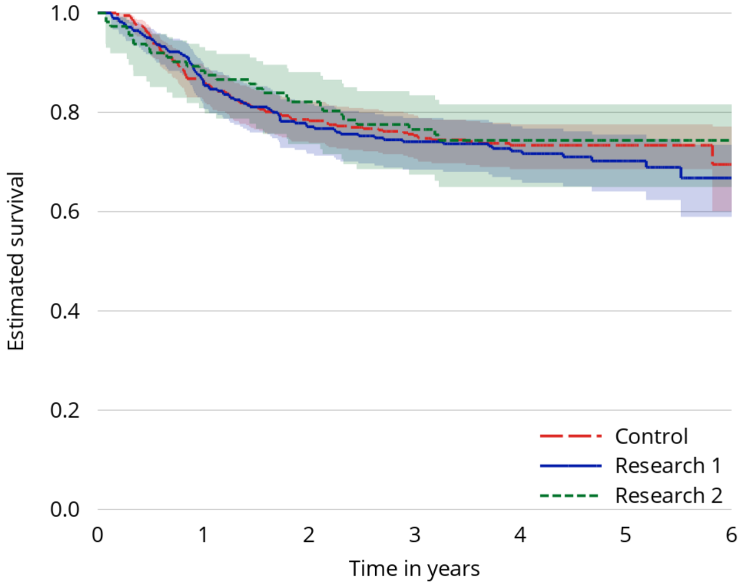

For peer review only - http://bmjopen.bmj.com/site/about/guidelines.xhtml

Figure 18: Fading of the Kaplan–Meier estimates to depict uncertainty (*LY09* trial). Here, the curves fade in proportion to the cumulative number of censored individuals (since it is censoring, not events, which means the estimate becomes more uncertain as time passes). The aim is to explicitly give the reader a visual deterrent when the eye is drawn to the far right.

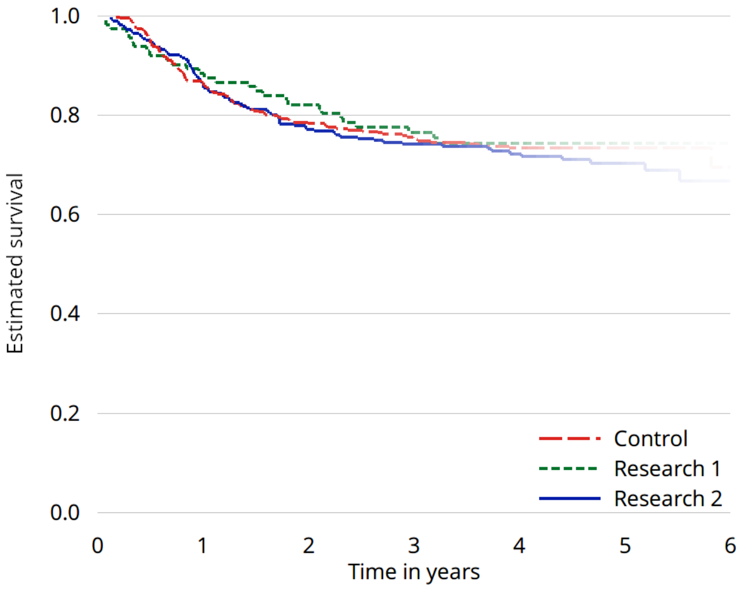

review only

For peer review only - http://bmjopen.bmj.com/site/about/guidelines.xhtml

1.4 Survey participants' experience with Kaplan–Meier

Figure 19: Years of experience 'reading and interpreting' *vs.* 'creating' Kaplan–Meier plots reported by participants. The margins give bar charts for 'reading and interpreting' (top) and 'creating' (right). For the bivariate plot, the top left indicates more time 'reading and interpreting' than 'creating' Kaplan–Meier plots.

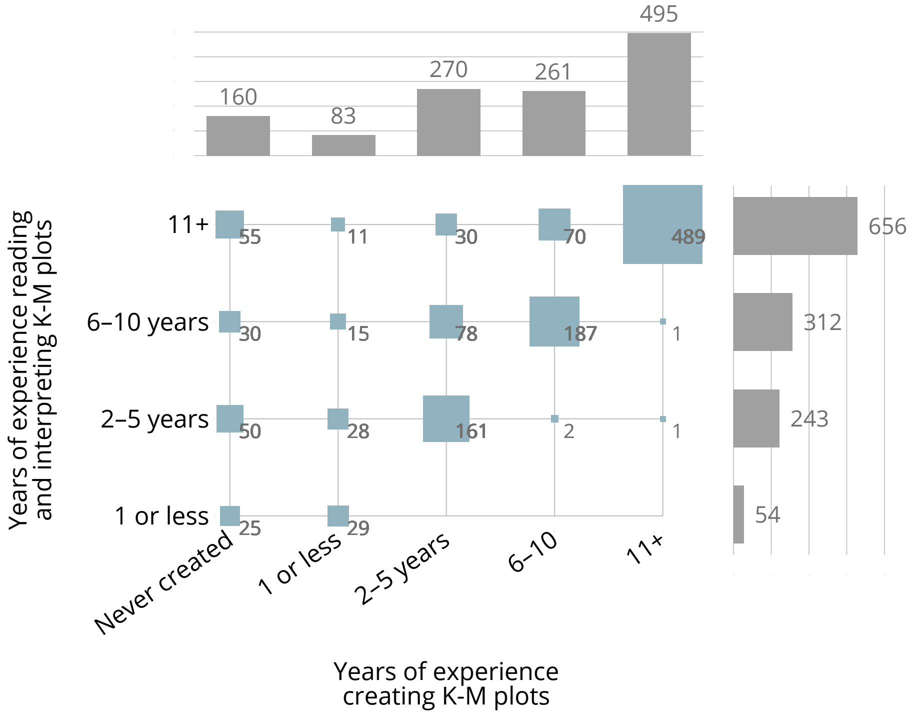

For peer review only - http://bmjopen.bmj.com/site/about/guidelines.xhtml

Figure 20: Left: Summary of the nature of free-text comments (not mutually exclusive) on the specific candidate graphs; Right: Comments, suggestions and improvements, either specific to a graph or left as a general comment

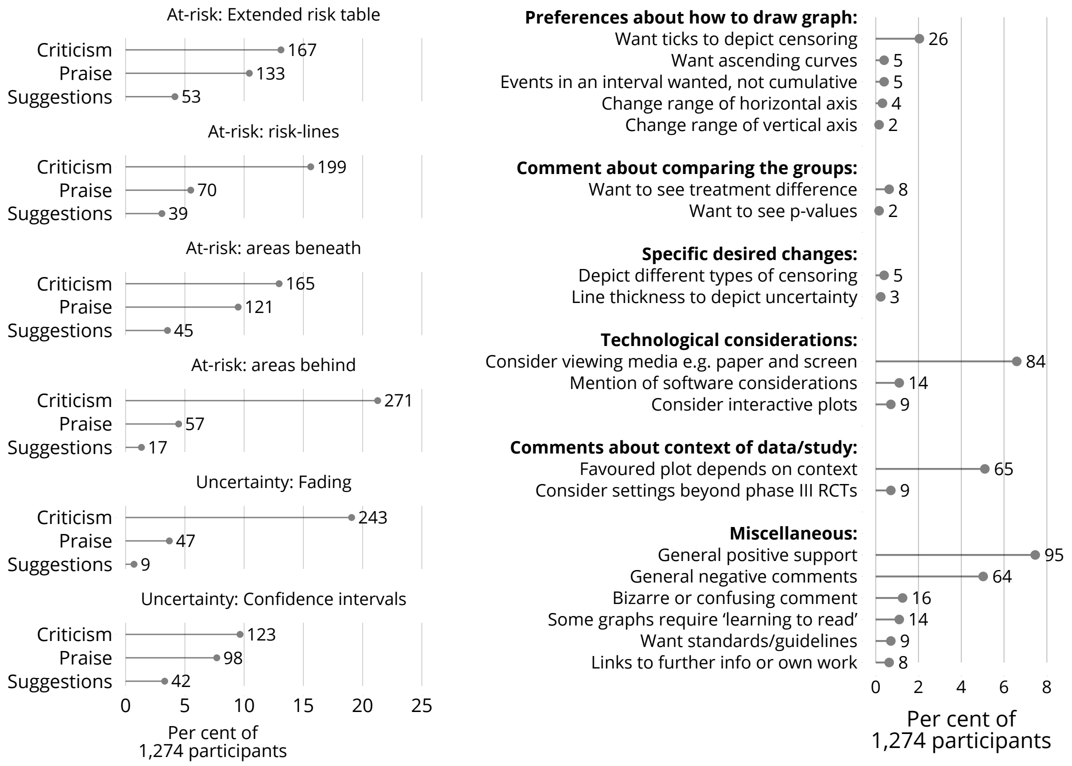

review only

For peer review only - http://bmjopen.bmj.com/site/about/guidelines.xhtml
